# Significance of the Diversification of Wheat Species for the Assembly and Functioning of the Root-Associated Microbiome

**DOI:** 10.3389/fmicb.2021.782135

**Published:** 2022-01-04

**Authors:** Cécile Gruet, Daniel Muller, Yvan Moënne-Loccoz

**Affiliations:** Univ Lyon, Université Claude Bernard Lyon 1, Centre National de la Recherche Scientifique (CNRS), Institut National de la Recherche pour l’Agriculture, l’Alimentation et l’Environnement (INRAE), VetAgro Sup, UMR 5557 Ecologie Microbienne, Villeurbanne, France

**Keywords:** wheat, domestication, rhizosphere, root microbiome, microbial interactions, symbiosis

## Abstract

Wheat, one of the major crops in the world, has had a complex history that includes genomic hybridizations between *Triticum* and *Aegilops* species and several domestication events, which resulted in various wild and domesticated species (especially *Triticum aestivum* and *Triticum durum*), many of them still existing today. The large body of information available on wheat-microbe interactions, however, was mostly obtained without considering the importance of wheat evolutionary history and its consequences for wheat microbial ecology. This review addresses our current understanding of the microbiome of wheat root and rhizosphere in light of the information available on pre- and post-domestication wheat history, including differences between wild and domesticated wheats, ancient and modern types of cultivars as well as individual cultivars within a given wheat species. This analysis highlighted two major trends. First, most data deal with the taxonomic diversity rather than the microbial functioning of root-associated wheat microbiota, with so far a bias toward bacteria and mycorrhizal fungi that will progressively attenuate thanks to the inclusion of markers encompassing other micro-eukaryotes and archaea. Second, the comparison of wheat genotypes has mostly focused on the comparison of *T. aestivum* cultivars, sometimes with little consideration for their particular genetic and physiological traits. It is expected that the development of current sequencing technologies will enable to revisit the diversity of the wheat microbiome. This will provide a renewed opportunity to better understand the significance of wheat evolutionary history, and also to obtain the baseline information needed to develop microbiome-based breeding strategies for sustainable wheat farming.

## Introduction

Plants interact with a myriad of microorganisms, and plant-microbe interactions are now considered a key facet of plant evolution, adaptation and ecology (Simon et al., 2019), both for wild and domesticated plants (Hassani et al., 2018). Hence, the plant needs to be seen as a holobiont (i.e., macro-organism and its associated microbiota), which requires a more integrated perspective on the significance of their microbial partners and the extended plant phenotypes they confer (Haichar et al., 2008; Vandenkoornhuyse et al., 2015).

**FIGURE 1:**
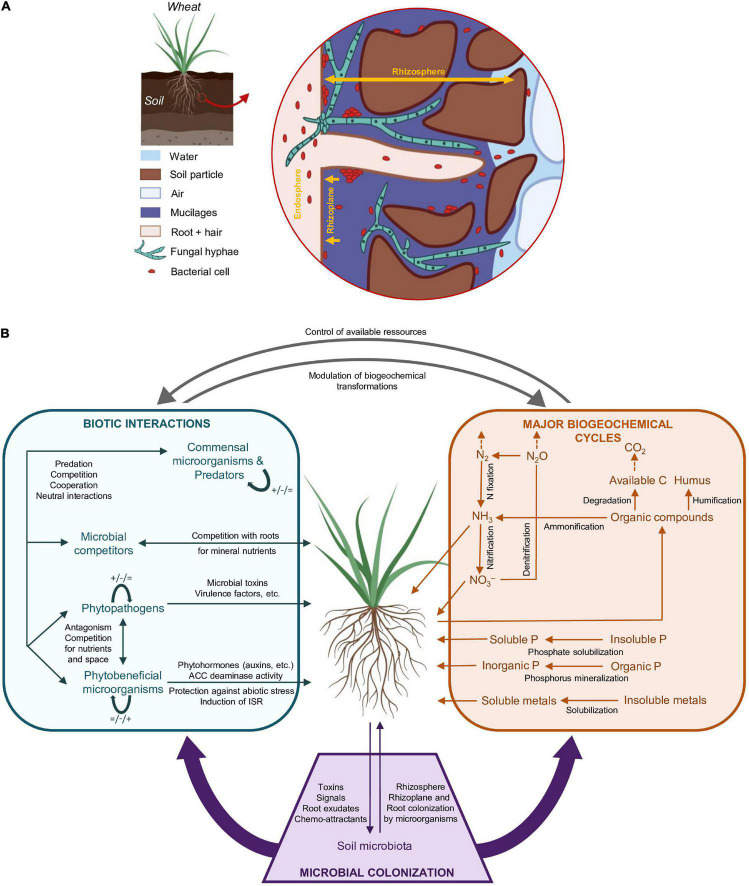
Relationship between wheat roots and soil/microbial components. **(A)** Structure of the rhizosphere, rhizoplane, and endosphere (not to scale). The rhizosphere is the soil in the immediate vicinity of the root, where the root has a major direct impact on soil organization and microbial functioning. The rhizoplane is the interface between the root surface and the soil. The endosphere corresponds to root internal tissues. Adapted from York et al. (2016) and Ding et al. (2019). **(B)** Major root-level microbial contributions to biotic interactions and biogeochemical cycles linked to plant growth and health. Root colonization by microorganisms is mediated by plant signals and exudates, which attract or repel soil microorganisms. Biotic interactions in the rhizosphere include plant-microorganism interactions and microorganism/microorganism interactions, with beneficial (+), deleterious (-) or neutral effects ( = ). Major microbial transformations are indicated for C, N, and P biogeochemical cycles. Metal biotransformations are not reviewed. A particular microbial taxon may be involved in several different biotic interactions (left box) and biotransformations (right box). ISR, Induced Systemic Resistance; ACC, 1-AminoCyclopropane-1-Carboxylate. Dashed arrows are used for abiotic volatilization phenomena.

The vast majority of plant microorganisms are in interaction with roots (Moënne-Loccoz et al., 2015). There are three distinct root-associated compartments for microorganisms, which are (i) the root endosphere (i.e., root internal tissues), (ii) the rhizoplane (i.e., the interface between the root surface and soil), and (iii) the rhizosphere (i.e., soil in the immediate vicinity of the root) (Figure 1A). Endophytic microorganisms inhabit the endosphere, where probably they have direct access to certain plant metabolites (Reinhold-Hurek and Hurek, 2011). They are often transmitted horizontally (Edwards et al., 2015), but some of them may be transmitted vertically (Liu et al., 2012; Hodgson et al., 2014; Truyens et al., 2015). Many of them if not most are thought to benefit their plant host (Schulz and Boyle, 2006; Reinhold-Hurek et al., 2015). In the rhizosphere, where soil is under the direct influence of the root (Hiltner, 1904), microorganisms from the surrounding soil are attracted by and benefit from rhizodeposits including root exudates (Zhalnina et al., 2018), leading to microbial proliferation and enhanced activity, i.e., the rhizosphere effect (Buée et al., 2009). Plant genotype influences the rhizosphere microbiota (Badri and Vivanco, 2009; Berg and Smalla, 2009; Micallef et al., 2009; Bouffaud et al., 2014), because different plant genotypes display different root properties and lead to different rhizosphere conditions for microbial partners. In turn, rhizosphere microorganisms can be either beneficial, pathogenic or have no effect on the plant (Vacheron et al., 2013; Nowell et al., 2016; Parnell et al., 2016). These plant-microbe interactions are essential for the ecological functioning of soil ecosystems (Lu et al., 2018).

Wheat, of the *Poaceae* family, is one of the major crops in the world with rice and maize. The crop provides 20% of calories in the human diet (Gill et al., 2004). Durum wheat is of significance as a food crop to make for example pasta, couscous, burghul, and bread wheat is used to prepare bread, pastries, etc. The Food and Agricultural Organization of the United Nations predicts a production of 776 million tons of wheat in 2022, an increase of 118 million compared to 2012. Demand for wheat is increasing with the change of diet in several large countries, such as China or India (Brisson et al., 2010). This increase in production needs to be achieved despite the growing number of challenges facing the crop, including climatic change, diminishing water resources, restrictions in the use of fertilizers and pesticides, and the risk caused by new and more aggressive pests (Tian et al., 2021). Intensive cereal systems for increasing yields are environmentally deleterious in the long-term (Lobell et al., 2009), and developing sustainable crops based on ecological intensification is essential. Exploiting the potential of wheat interactions with soil microorganisms that can enhance plant productivity, by contributing to plant nutrition and health (Bhattacharyya and Jha, 2012; Vacheron et al., 2013) is a promising strategy to reach this goal. This will require a better, more comprehensive understanding of the microbial community associated with wheat, and to identify new avenues to exploit them for sustainable wheat farming.

Recent methodology improvements, especially in sequencing technologies, have enabled to revisit our knowledge of the interactions between wheat and root-associated microbial community. For instance, the wheat microbiome has been recently described, with a focus on environmental factors driving microbiome assembly and identifying beneficial microorganisms important for sustainable wheat farming (Kavamura et al., 2021). This review aims at putting into perspective the growing knowledge on wheat-microbe interactions, by considering the evolutionary history of wheats and then its implications for the wheat microbiome. The particular patterns of microbial selection in the different root compartments (rhizosphere, rhizoplane, and endosphere) are described, ranging from bacteria and archaea to fungi and other microeukaryotes. Finally, we focus on the functional diversity of the wheat root microbiome and its implication for wheat growth and health.

## Wheat Particularities of Relevance for Plant-Microbe Interactions

### Hybridization, Polyploidy, and Domestication History

**FIGURE 2:**
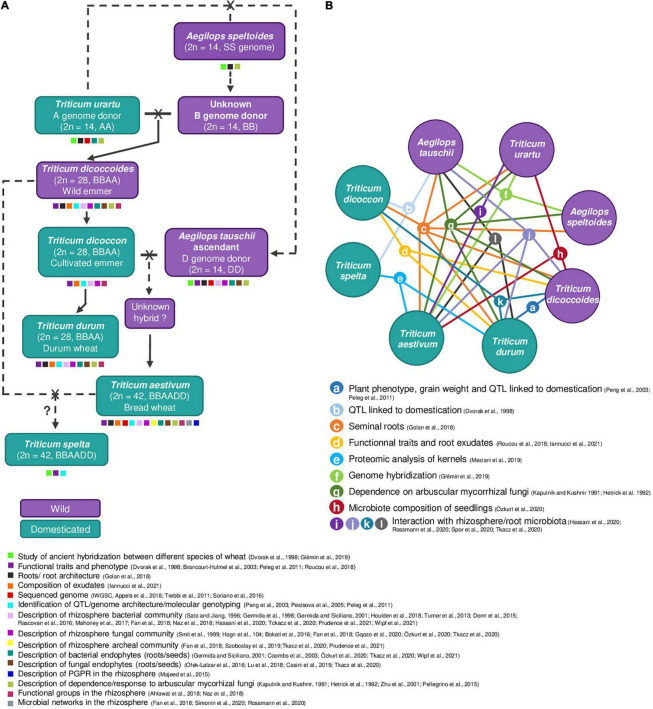
The origin of durum and bread wheat, and literature comparisons. Wild wheats are represented in purple, while domesticated wheats are in turquoise. **(A)** Wild and domesticated species involved in wheat evolution and leading to pasta (*T. durum*) and bread (*T. aestivum*) wheats are indicated (adapted from Mujeeb-Kazi, 2006), as well as examples of key scientific issues investigated with them (shown with small squares with the color code indicated below the panel). The ancestors of pasta and bread wheats underwent hybridization and polyploidization events involving genomes A, S, B, and D. A simplified version of wheat evolutionary history is depicted. The A and S genomes arose by divergence from a common ancestor (not shown) circa 7 million years Before Present (BP) (Pont et al., 2019). The B genome probably descends from the S genome and is therefore a close relative of *Aegilops speltoides* (SS) (Fricano et al., 2014). A first hybridization event is speculated to have taken place between A (*T. urartu*) and S (*A. speltoides*/*A. mutica*) genomes, 5–6 million years BP (Glémin et al., 2019), leading to the D genome upon homoploid hybrid speciation. A second hybridization event took place about 500,000 years BP between this B genome donor and *T. urartu* (A genome), leading to the wild tetraploid *T. dicoccoides*, and later to the domesticated emmer *T. dicoccon*. A third hybridization event (10,000 years BP) involved *T. dicoccon* and an ascendant of current *A. tauschii* (D genome), leading to the hexaploid wheat *T. aestivum*. It is unclear whether the latter hybridization and domestication events took place at the same time or not, and the wild form of the hexaploid hybrid remains unknown. A fourth cross, between *T. aestivum* and *T. dicoccon*, is probably at the origin of the hexaploid wheat *Triticum spelta* (Fricano et al., 2014). Wheat genomes are composed of 14 (AA, BB or DD), 28 (AABB), or 42 chromosomes (AABBDD). Dashed arrows are used for uncertain events. In the history of Triticeae, other domestication events also occurred but without leading to species extensively cultivated nowadays, as for example the wild einkorn *Triticum monococcum* subsp. *beoticum* (A genome, genomically close to but not interfertile with *T. urartu*; Fricano et al., 2014) was domesticated to become *Triticum monococcum* subsp. *monococcum* (not shown). **(B)** Key literature comparisons between individual wheat species are indicated using colored lines connecting the corresponding species included; the type of comparison is shown using letters a-l, and is specified in the legend, along with the corresponding reference(s). The figure points to an unbalance in the consideration of wheat species, as previous investigation have studied *T. durum*, *T. aestivum*, and *T. dicoccoides* extensively, *T. urartu*, *T. dicoccon*, and *A. tauschii* to a lesser extent, but the other species have been seldom considered. We identified eight studies comparing wheat genomic and phenotypic properties and seven others comparing the microbiota associated to different wheat species, which shows that plant properties and microbiota properties are described to the same extent. Multiple comparisons between *T. durum*, *T. dicoccon*, and *T. dicoccoides* were made (five studies), probably because this represents a good model for domestication studies, but only one considered the microbiota (h). Only 3 of 15 studies, with a focus on seminal roots (c) or arbuscular mycorrhizal fungi (g), covered all main events of wheat history.

The *Triticum* and *Aegilops* ancestors of bread wheat (*Triticum aestivum*) and durum wheat (*Triticum durum*) underwent hybridization, as well as polyploidization events (Haberer et al., 2016) involving genomes A, S, B and D (Figure 2A). The A and S genomes arose by divergence from a common ancestor circa 7 million years Before Present (BP) (Pont et al., 2019). D genome might have originated from homoploid hybrid speciation of A and S genomes, 5–6 million years BP (Glémin et al., 2019). Two wild diploid wheats (2*n* = 14), i.e., *Triticum urartu* (AA genome) and a close descendant of *Aegilops speltoides* (BB genome) (Pont et al., 2019), hybridized about 500,000 years BP and gave a tetraploid wild wheat (2*n* = 28) termed *Triticum dicoccoides* (wild emmer wheat; AABB genome) (Pont et al., 2019). A second hybridization took place about 10,000 years BP, between domesticated emmer and a direct ascendant of the current diploid species *Aegilops tauschii* (DD genome), giving rise to a wild hexaploid wheat (2*n* = 42; AABBDD genome) at the origin of domesticated *T. aestivum*. Hexaploid wheat might have arisen from more than one crossing event (Dvorak et al., 1998). In both hybridization events, the seven chromosomes of each genome (A, B, or D) could not pair for subsequent mitosis, which resulted in chromosome doubling and thus allopolyploidy (Glover, 2016). On one hand, hybridization can lead to a loss of genetic diversity, since only a limited number of individuals of each species is involved in the crossing. On the other hand, polyploidy may lead to particular gene expression patterns, and probably also to particular properties in terms of root exudation, root uptake, etc. (Saia et al., 2019; Iannucci et al., 2021), which can be expected to impact on microorganisms.

Wheat has undergone several domestication events. The wild einkorn *Triticum monococcum* subsp. *beoticum* (genomically close to *T. urartu*; Fricano et al., 2014) was domesticated and gave *T. monococcum* subsp. *monococcum*, a crop seldom cultivated nowadays (Salamini et al., 2002) (and therefore is not portrayed in Figure 2A). The wild emmer wheat *T. dicoccoides* (AABB genome) gave rise to the domesticated emmer wheat *T. dicoccon* (perhaps on several independent occasions (Özkan et al., 2011)), which later evolved into durum wheat *T. durum* (Figure 2A). The cross between wild emmer and the ascendant of *A. tauschii* (DD genome) either (i) resulted in an unknown hexaploid wild wheat, from which derived the domesticated wheat *T. aestivum* (Salamini et al., 2002), or (ii) was concomitant with the domestication event itself. Agronomic traits of wheat changed gradually upon domestication. As for other *Poaceae*, domesticated wheat presents bigger grains and higher seed number per spike (Salamini et al., 2002). Wheat domestication resulted also in lower root biomass (Waines and Ehdaie, 2007), with more fine roots and a shallower root system (Roucou et al., 2018), and with more seminal roots (Golan et al., 2018), but these traits display heterogeneity at inter and infra-species levels. Domestication represents a genetic bottleneck, with an estimated 50–60% reduction of wheat genetic diversity (Bonnin et al., 2014).

### Wheat Geography

Nowadays, wild wheat habitats are still located in the area that was 10,000 years ago the Fertile Crescent (Supplementary Figure 1), and where *T. urartu*, *T. beoticum*, *T. dicoccoides*, *A. speltoides*, and *A. tauschii* can be found. Beyond the Fertile Crescent, wild wheats grow mainly in temperate climates between latitudes 30°N and 40°N, but they may occur also within the Arctic Circle and to higher elevations near the equator (Haas et al., 2019). Therefore, wild wheats grow under a wide range of pedoclimatic conditions, which means they may encounter different types of soil microbial communities.

Whereas wild wheats are mostly winter type, domesticated wheat can be either winter or spring type (i.e., does not need vernalization), which enables to find domesticated wheat in a larger range of climatic zones (and soil conditions). In comparison with spring wheat, winter wheat is sown in the autumn, which means the root system develops and interact with soil microorganisms over a much longer duration in the year. Domesticated wheats (both durum and bread wheats) are found in a broad range of areas and climates, and are present on all continents (Di Paola et al., 2018; Mideksa et al., 2018; Dong et al., 2019; Tidiane Sall et al., 2019). They are therefore likely to be included in a diversity of cropping systems and farming practices (i.e., regarding tillage, fertilizers, etc.) in contrasted conditions of soil and climate, which means exposure to very different types of soil microbial communities (Chaudhary et al., 2017; Dong et al., 2017; Gajda et al., 2017; Somenahally et al., 2018; Wang et al., 2018).

### Selection and Modern Breeding

Growth of domesticated wheats in diverse environments and climatic conditions required local adaptations (Dwivedi et al., 2016). Farmers, through mass selection, led to the creation of particular, still genetically heterogeneous (Bonjean, 2001) wheat genotypes (called landraces) well-adapted to local environments (Kiszonas and Morris, 2018) and to specific stresses (Feldman and Kislev, 2007). Thus, the growth of landraces results in stands consisting of mixtures of many different closely related genotypes. Considering features important for plant-microbe interactions, this means an expected heterogeneity in terms of root system traits, plant physiology, rhizodeposition patterns and rhizosphere chemistry within a given plot.

Modern breeding aimed at higher yield. Genealogical selection (Gayon and Zallen, 1998) resulted into (i) limited plant-to-plant genetic heterogeneity within these cultivars, (ii) preferential allocation of N and C compounds to shoots rather than roots, probably leading to reduced rhizodeposition for microorganisms (Lindig-Cisneros et al., 1997), (iii) enhanced mineral uptake (Zhang et al., 2020; Cantarel et al., 2021), and (iv) particularities in root functioning and rhizosphere chemistry (George et al., 2014).

During the Green Revolution (from 1950 to late 1960s), crosses with semi-dwarf varieties were implemented (Brancourt-Hulmel et al., 2003). Hybrids were produced (Šramková et al., 2009). Chromosome engineering methodologies have been employed to transfer specific disease genes from other members of the tribe Triticeae into wheat, conferring new immune system defenses against phytopathogens (Rong et al., 2000; Niu et al., 2011). More recently, molecular markers and quantitative trait loci (QTLs) (Peng et al., 2003; Pestsova et al., 2005; Peleg et al., 2011) have been used successfully to facilitate breeding, whereas CRISPR-Cas9 (Kiszonas and Morris, 2018) and genome sequencing (Trebbi et al., 2011; Jia et al., 2013; Ling et al., 2013; Maccaferri et al., 2014; Soriano et al., 2016; International Wheat Genome Sequencing Consortium [IWGSC], Appels et al., 2018) open new perspectives, with potentially an impact on wheat-microbe interactions.

## Taxonomic Diversity of Microorganisms in the Rhizosphere and Roots of Wheat

### Importance and Analysis of Root-Associated Wheat Microbiome

Soil type (Donn et al., 2015; Simonin et al., 2020) as well as cultivation history (Hilton et al., 2018) and practices (e.g., tillage, soil amendments) are the main factors shaping wheat root microbiota (Ahlawat et al., 2018; Kavamura et al., 2018). The second most important factor is the wheat genotype, both at the species and intra-species (varieties) levels (Mahoney et al., 2017; Stromberger et al., 2017; Ellouze et al., 2018; Naz et al., 2018; Kinnunen-Grubb et al., 2020; Iannucci et al., 2021). Growth stage and plant physiology matter less (Houlden et al., 2008; Donn et al., 2015), despite significant shifts after tillering (Wang J. et al., 2016) and when heading starts (Hilton et al., 2018).

In the case of wheats (Triticeae tribe), most work on rhizosphere and root microbiomes has focused on bread wheat *T. aestivum*, whereas durum wheat *T. durum* has received little attention (Figure 2B). Therefore, knowledge on other *Triticum* species and *Aegilops* species is very incomplete (Özkurt et al., 2020; Tkacz et al., 2020).

Other species of the Triticeae tribe have been considered mostly for comparison with *T. aestivum* and *T. durum*, to decipher the impact of domestication and selection on the wheat microbiome (Golan et al., 2018; Roucou et al., 2018; Meziani et al., 2019) (Figure 2B). For instance, the interactions with *Glomeromycota* fungi have been compared between wild (*T. urartu, A. speltoides*, etc.) and domesticated wheats (*T. aestivum*, etc.) (Kapulnik and Kushnir, 1991; Hetrick et al., 1992).

Microorganisms are subjected to stronger plant selection in the root endosphere than the rhizosphere (Compant et al., 2010; Fitzpatrick et al., 2018), including in the case of wheat. This materializes by greater dominance effects in the wheat endosphere, i.e., with fewer taxa but in greater relative abundance, both for bacteria and fungi (Lu et al., 2018; Özkurt et al., 2020; Tkacz et al., 2020; Prudence et al., 2021). While the rhizosphere was extensively studied, fewer studies have focused on the root endosphere of wheats (Germida and Siciliano, 2001; Bokati et al., 2016; Özkurt et al., 2020; Tkacz et al., 2020). In addition, the bacterial community has been more investigated than the archaeal and fungal communities. Information about the archaeal community of wheat rhizosphere and root endosphere exists almost only for *T. aestivum*, and this community has been documented by culture-independent methods only (Fan et al., 2018; Szoboszlay et al., 2019; Tkacz et al., 2020; Prudence et al., 2021), archaea being difficult to isolate with methods routinely used in rhizosphere ecology. Very few studies have focused on fungi in the root endosphere, whether with culture-dependent (Bokati et al., 2016) or culture-independent methods (Bokati et al., 2016; Ofek-Lalzar et al., 2016; Özkurt et al., 2020). It must be kept in mind that the various root-associated compartments, i.e., root endosphere, rhizoplane and rhizosphere, are not always straightforward to distinguish from one another from an experimental point of view, which complicates comparisons between studies.

### Rhizosphere, Rhizoplane, and Root Endosphere Microbiomes of Bread Wheat

#### Rhizosphere Microbiome

The rhizosphere bacterial community of *T. aestivum* is dominated at almost 40% by *Proteobacteria*, as indicated by culture-independent methods (Table 1). The other dominant phyla (10–15%) are *Acidobacteria*, *Actinobacteria*, and *Bacteroidetes*, whereas the remaining phyla represent <5% each (Turner et al., 2013; Donn et al., 2015; Rascovan et al., 2016; Mahoney et al., 2017; Fan et al., 2018; Tkacz et al., 2020; Prudence et al., 2021). Culture-dependent methods point to *Proteobacteria* and *Actinobacteria* (each representing about 25%) and then *Firmicutes* (10%) as main phyla in the *T. aestivum* rhizosphere (Table 2; Juhnke et al., 1987; Sato and Jiang, 1996a,b; Germida et al., 1998; Germida and Siciliano, 2001). Within the *Proteobacteria*, the *Gammaproteobacteria* are the most abundant, with especially the families *Pseudomonadaceae* and *Xanthomonadaceae* (Donn et al., 2015).

**TABLE 1:** Occurrence of phyla in the rhizosphere of *T. aestivum*, as documented by culture-independent methods.

Phyla	Occurrence (%)^a^			Number (and list^b^) of *T. aestivum* genotypes	Number of countries	References
	Min	Max	Mean			
**Bacteria**						
*Proteobacteria*	16.5 (Bawburgh, UK; a)	52.0 (Villa Saboya, Argentina; l)	38.6	14 (a, b, c, d, e, f, g, h, i, j, k, l, NS)	5	Turner et al., 2013; Donn et al., 2015; Rascovan et al., 2016; Mahoney et al., 2017; Fan et al., 2018; Tkacz et al., 2020; Prudence et al., 2021
*Acidobacteria*	0.5 (Gundibinyal, Australia; k)	23.4 (Bawburgh, UK; a)	11.5	14 (a, b, c, d, e, f, g, h, i, j, k, l, NS)	5	Turner et al., 2013; Donn et al., 2015; Rascovan et al., 2016; Mahoney et al., 2017; Fan et al., 2018; Tkacz et al., 2020; Prudence et al., 2021
*Actinobacteria*	1.0 (Villa Saboya, Argentina; l)	26.0 (Gundibinyal, Australia; k)	12.9	14 (a, b, c, d, e, f, g, h, i, j, k, l, NS)	5	Turner et al., 2013; Donn et al., 2015; Rascovan et al., 2016; Mahoney et al., 2017; Fan et al., 2018; Tkacz et al., 2020; Prudence et al., 2021
*Bacteroidetes*	0.9 (Villa Saboya, Argentina; l)	28.0 (Pullman, WA; g)	14.6	14 (a, b, c, d, e, f, g, h, i, j, k, l, NS)	5	Turner et al., 2013; Donn et al., 2015; Rascovan et al., 2016; Mahoney et al., 2017; Fan et al., 2018; Tkacz et al., 2020; Prudence et al., 2021
*Chloroflexi*	0.5 (Northern China; NS)	6.3 (Bawburgh, UK; a)	0.9	7 (a, k, l, NS)	4	Donn et al., 2015; Fan et al., 2018; Tkacz et al., 2020; Prudence et al., 2021
*Cyanobacteria*	0.5 (Norwich, UK; a)	3.0 (Bawburgh, UK; a)	0.2	1 (a)	2	Turner et al., 2013; Tkacz et al., 2020
*Firmicutes*	9.6 (Norwich, UK; a)	20.9 (Bawburgh, UK; a)	2.9	2 (a, NS)	3	Turner et al., 2013; Rascovan et al., 2016; Prudence et al., 2021
*Armatimonadetes*	1.0 (Pullman, WA; d)	4.6 (Pullman, WA; e)	1.2	9 (b, c, d, e, f, g, h, i, j)	1	Mahoney et al., 2017
*Planctomycetes*	0.2 (Northern China; NS)	8.9 (Villa Saboya, Argentina; l)	1.0	4 (a, h, l, NS)	3	Turner et al., 2013; Rascovan et al., 2016; Mahoney et al., 2017; Fan et al., 2018
*Saccharibacteria*	2.0 (Pullman, WA; i)	4.0 (Pullman, WA; b)	2.0	9 (b, c, d, e, f, g, h, i, j)	1	Mahoney et al., 2017
*Gemmatimonadetes*	0.5 (Northern China; NS)	7.0 (Pullman, WA; b)	2.3	10 (b, c, d, e, f, g, h, i, j, NS)	2	Fan et al., 2018; Naz et al., 2018
*Verrucomicrobia*	0.7 (Northern China; NS)	10.2 (Villa Saboya, Argentina; l)	2.7	12 (a, b, c, d, e, f, g, h, i, j, l, NS)	4	Turner et al., 2013; Rascovan et al., 2016; Mahoney et al., 2017; Tkacz et al., 2020; Prudence et al., 2021
Other taxa	2.0 (Villa Saboya, Argentina; l)	23.5 (Bawburgh, UK; a)	2.2	2 (a, l)	3	Turner et al., 2013; Rascovan et al., 2016; Prudence et al., 2021
Unidentified taxa	1.0 (Pullman, WA; e)	71.6 (Northern China; NS)	7.12	11 (b, c, d, e, f, g, h, i, j, k)	2	Donn et al., 2015; Mahoney et al., 2017
**Archaea**						
*Euryarchaeota*	24.5 (Bawburgh, UK; a)	24.5 (Bawburgh, UK; a)	6.1	1 (a)	1	Prudence et al., 2021
*Thaumarchaeota*	22.2 (Northern China; NS)	100.0 (Bawburgh, UK; a)	74.3	3 (a, NS)	3	Fan et al., 2018; Szoboszlay et al., 2019; Tkacz et al., 2020; Prudence et al., 2021
Other taxa	0.2 (Bawburgh, UK; a)	0.2 (Bawburgh, UK; a)	0.05	1 (a)	1	Prudence et al., 2021
Unidentified taxa	0.4 (Bawburgh, UK; a)	77.8 (Northern China; NS)	19.6	2 (a, NS)	2	Fan et al., 2018; Prudence et al., 2021
**Fungi**						
*Zygomycota*	16.0 (Bawburgh, UK; a)	16.0 (Bawburgh, UK; a)	2.7	1 (a)	1	Tkacz et al., 2020
*Glomeromycota*	0.5 (Bethlehem, South Africa; o)	1.5 (Bawburgh, UK; a)	0.8	4 (a, m, n, o)	2	Gqozo et al., 2020; Tkacz et al., 2020
*Chytridiomycota*	0.9 (Bawburgh, UK; a)	22.3 (Pretoria, South Africa; n)	5.4	4 (m, n, o, p)	2	Gqozo et al., 2020; Tkacz et al., 2020
*Basidiomycota*	0.5 (Hangzhou, China; NS)	24.3 (Bethlehem, South Africa; o)	12.6	5 (a, m, n, o, NS)	3	Smit et al., 1999; Lu et al., 2018; Tkacz et al., 2020
*Ascomycota*	28.3 (Bawburgh, UK; a)	60.6 (Napier, South Africa; m)	45.4	6 (a, m, n, o, NS)	3	Fan et al., 2018; Lu et al., 2018; Gqozo et al., 2020; Tkacz et al., 2020
Other taxa	12.6 (Bethlehem, South Africa; o)	39.5 (Bawburgh, UK; a)	14.2	4 (a, m, n, NS)	2	Gqozo et al., 2020; Tkacz et al., 2020
Unidentified taxa	51.0 (Northern China; NS)	63.5 (Hangzhou, China; NS)	19.1	2 (NS)	1	Lu et al., 2018

*Min and Max correspond to minimum and maximum occurrences observed for each phylum in the specified references, after combining data from all replicate plants of one genotype (indicated between parentheses), at one growth stage, for one treatment in one soil of one geographic location (indicated between parentheses). For each phylum, the mean is calculated from all the values obtained from the different geographic locations and T. aestivum genotypes.*

*^a^Abbrevations are used to designate United Kingdom (UK) and Washington State (WA).*

*^b^Genotypes were cultivars Paragon (a), Madsen (b), PI561725 (c), Eltan (d), Finch (e), Hill81 (f), Lewjain (g), PI561722 (h), PI561726 (i), PI561725 (j), Janz (k), Cadenza (l), SST88 (m), Kariega (n), Eland (o) or were not specified (NS).*

**TABLE 2:** Occurrence of phyla in the rhizosphere of *T. aestivum*, as documented by culture-dependent methods.

Phyla	Occurrence (%)			Number (and list^a^) of *T. aestivum* genotypes	Number of countries	References
	Min	Max	Mean			
**Bacteria**						
*Proteobacteria*	9.4 (Watrous, Canada; NS)	77.0 (Kawatabi, Japan; d)	23.7	6 (a, b, c, d, f, NS)	3	Sato and Jiang, 1996b; Germida et al., 1998; Germida and Siciliano, 2001; Rascovan et al., 2016
*Actinobacteria*	5.0 (Watrous, Canada; NS)	88.9 (Kawatabi, Japan; d)	24.0	6 (a, b, c, d, f, NS)	3	Juhnke et al., 1987; Sato and Jiang, 1996a; Germida et al., 1998; Germida and Siciliano, 2001
*Bacteroidetes*	1.9 (Watrous, Canada; NS)	23.0 (Kawatabi, Japan; d)	5.9	5 (a, b, c, d, NS)	2	Sato and Jiang, 1996b; Germida et al., 1998; Germida and Siciliano, 2001
*Firmicutes*	3.4 (Saskatoon, Canada; b)	28.3 (Watrous, Canada; NS)	10.0	5 (a, b, c, d, NS)	2	Sato and Jiang, 1996a,b; Germida et al., 1998; Germida and Siciliano, 2001
Other taxa	55.4 (Watrous, Canada; NS)	55.4 (Watrous, Canada; NS)	7.9	1 (NS)	1	Germida et al., 1998
Unidentified taxa	60.6 (Saskatoon, Canada; a)	70.0 (Saskatoon, Canada; c)	28.5	3 (a, b, c)	1	Germida and Siciliano, 2001
**Fungi**						
*Zygomycota*	5.9 (Utrecht, The Netherlands; NS)	5.9 (Utrecht, The Netherlands; NS)	5.9	1 (NS)	1	Smit et al., 1999
*Chytridiomycota*	3.7 (Utrecht, The Netherlands; NS)	6.7 (Utrecht, The Netherlands; NS)	3.7	1 (NS)	1	Smit et al., 1999
*Basidiomycota*	32.8 (Utrecht, The Netherlands; NS)	32.8 (Utrecht, The Netherlands; NS)	32.8	1 (NS)	1	Smit et al., 1999
*Ascomycota*	54.6 (Utrecht, The Netherlands; NS)	54.6 (Utrecht, The Netherlands; NS)	54.6	1 (NS)	1	Smit et al., 1999
Other taxa	3.7 (Utrecht, The Netherlands; NS)	6.7 (Utrecht, The Netherlands; NS)	3.7	1 (NS)	1	Smit et al., 1999

*Min and Max correspond to minimum and maximum occurrences observed for each phylum in the specified references, after combining data from all replicate plants of one genotype (indicated between parentheses), at one growth stage, for one treatment in one soil of one geographic location (indicated between parentheses). For each phylum, the mean is calculated from all the values obtained from the different geographic locations and T. aestivum genotypes.*

*^a^Genotypes were cultivars PI167549 (a), Red Fife (b), CDC Teal (c), Aoba (d), GSTR 11562 (e), Pondera (f), or not specified (NS).*

**FIGURE 3:**
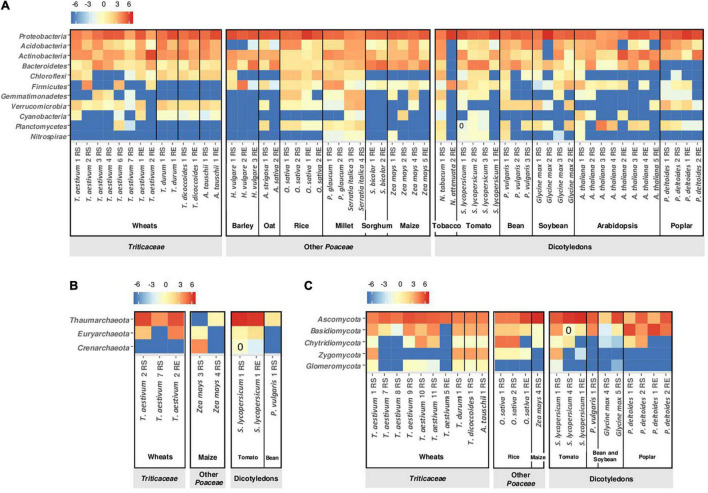
Heatmap of major phyla affiliated with **(A)** bacteria, **(B)** archaea, and **(C)** fungi in rhizosphere soil (RS) and root/endosphere (RE) of wheats and non-wheat plants based on results from selected studies. Only phyla with relative abundance >0.5% in at least one study are shown. The color intensity in each cell denotes the transformed relative abundance [log_2_((100x)+0.02)] of a phylum in each study for each plant type. For details on individual conditions, see Supplementary Table 1.

In the *archaea*, the *Thaumarchaeota* represent more than two thirds of the rhizosphere community of *T. aestivum*, the *Euryarchaeota* <10%, and a range of unidentified phyla a total of about 20% (Table 1; Fan et al., 2018; Szoboszlay et al., 2019; Tkacz et al., 2020; Prudence et al., 2021). In the studies cited in Table 1 and Figure 3B, the *Crenarchaeota* were not detected in *T. aestivum* rhizosphere. One investigation also considered lower taxonomic levels, showing that the *Nitrosphaeraceae* (*Thaumarchaeota*) was the most abundant family in the rhizosphere (Prudence et al., 2021).

The rhizosphere fungal community of *T. aestivum* is dominated by *Ascomycota*, which represent 40–50% of the total community with culture-independent (Table 1; Fan et al., 2018; Lu et al., 2018; Gqozo et al., 2020; Tkacz et al., 2020) and culture-dependent methods (Table 3; Smit et al., 1999; Hagn et al., 2003). The other dominant phyla are *Basidiomycota* and *Chytridiomycota* (5–15% each; Table 1). At genus level, *Mortiella* (phylum *Mucoromycota*), *Verticillum* (*Ascomycota*), and *Cryptococcus* (*Basidiomycota*) are enriched in the rhizosphere (Tkacz et al., 2020).

**TABLE 3:** Occurrence of phyla in the root endosphere of *T. aestivum*, as documented by culture-independent and culture-dependent methods.

Phyla	Occurrence (%)^a^			Number (and list^b^) of *T. aestivum* genotypes	Number of countries	References
	Min	Max	Mean			
**Culture-independent methods**						
**Bacteria**						
*Proteobacteria*	16.1 (Bawburgh, UK; a)	51.1 (Bawburgh, UK; a)	33.6	1 (a)	1	Tkacz et al., 2020; Prudence et al., 2021
*Acidobacteria*	1.2 (Bawburgh, UK; a)	12.4 (Bawburgh, UK; a)	6.8	1 (a)	1	Tkacz et al., 2020; Prudence et al., 2021
*Actinobacteria*	14.9 (Bawburgh, UK; a)	60.1 (Bawburgh, UK; a)	37.5	1 (a)	1	Tkacz et al., 2020; Prudence et al., 2021
*Bacteroidetes*	5.1 (Bawburgh, UK; a)	11.1 (Bawburgh, UK; a)	8.1	1 (a)	1	Tkacz et al., 2020; Prudence et al., 2021
*Chloroflexi*	1.0 (Bawburgh, UK; a)	5.2 (Bawburgh, UK; a)	3.1	1 (a)	1	Tkacz et al., 2020; Prudence et al., 2021
*Cyanobacteria*	4.3 (Bawburgh, UK; a)	4.3 (Bawburgh, UK; a)	2.2	1 (a)	1	Tkacz et al., 2020
*Firmicutes*	3.5 (Bawburgh, UK; a)	3.5 (Bawburgh, UK; a)	1.6	1 (a)	1	Prudence et al., 2021
*Verrucomicrobia*	7.17 (Bawburgh, UK; a)	7.17 (Bawburgh, UK; a)	3.6	1 (a)	1	Tkacz et al., 2020
Other taxa	7.0 (Bawburgh, UK; a)	7.0 (Bawburgh, UK; a)	3.5	1 (a)	1	Prudence et al., 2021
**Archaea**						
*Euryarchaeota*	28.0 (Bawburgh, UK; a)	28.0 (Bawburgh, UK; a)	28.0	1 (a)	1	Prudence et al., 2021
*Thaumarchaeota*	62.0 (Bawburgh, UK; a)	62.0 (Bawburgh, UK; a)	62.0	1 (a)	1	Prudence et al., 2021
Other taxa	7.9 (Bawburgh, UK; a)	7.9 (Bawburgh, UK; a)	7.9	1 (a)	1	Prudence et al., 2021
Unidentified taxa	2.1 (Bawburgh, UK; a)	2.1 (Bawburgh, UK; a)	2.1	1 (a)	1	Prudence et al., 2021
**Fungi**						
*Ascomycota*	66.0 (Kirksville, MO; b)	99.3 (Northern Germany; NS)	82.7	1 (b, NS)	1	Bokati et al., 2016
*Basidiomycota*	0.7 (Northern Germany; NS)	0.7 (Northern Germany; NS)	0.7	1 (NS)	1	Özkurt et al., 2020
*Other taxa*	34.0 (Kirksville, MO; b)	34.0 (Kirksville, MO; b)	17.0	1 (b)	1	Bokati et al., 2016
**Culture-dependent methods**						
**Bacteria**						
*Proteobacteria*	17.8 (Saskatoon, Canada; c)	24.3 (Saskatoon, Canada; e)	21.0	3 (c, d, e)	1	Germida and Siciliano, 2001
*Actinobacteria*	4.7 (Saskatoon, Canada; c)	11.8 (Saskatoon, Canada; e)	8.0	3 (c, d, e)	1	Germida and Siciliano, 2001
*Bacteroidetes*	1.0 (Saskatoon, Canada; d)	4.1 (Saskatoon, Canada; c)	2.5	3 (c, d, e)	1	Germida and Siciliano, 2001
*Firmicutes*	0.8 (Saskatoon, Canada; e)	1.6 (Saskatoon, Canada; c)	1.1	3 (c, d, e)	1	Germida and Siciliano, 2001
Unidentified taxa	59.0 (Saskatoon, Canada; e)	73.6 (Saskatoon, Canada; c)	67.3	3 (c, d, e)	1	Germida and Siciliano, 2001
**Fungi**						
*Ascomycota*	75.0 (Kirksville, MO; b)	75.0 (Kirksville, MO; b)	75.0	1 (b)	1	Bokati et al., 2016
Unidentified taxa	15.0 (Kirksville, MO; b)	15.0 (Kirsville, MO; b)	15.0	1 (b)	1	Bokati et al., 2016

*Min and Max correspond to minimum and maximum occurrences observed for each phylum in the specified references, after combining data from all replicate plants of one genotype (indicated between parentheses), at one growth stage, for one treatment in one soil of one geographic location (indicated between parentheses). For each phylum, the mean is calculated from all the values obtained from the different geographic locations and T. aestivum genotypes.*

*^a^Abbrevations are used to designate Missouri (MO) and United Kingdom (UK).*

*^b^Genotypes were cultivars Paragon (a), GSTR 11562 (b), PI167549 (c), Red Fife (d), CDC Teal (e) or not specified (NS).*

#### Rhizoplane Microbiome

The rhizoplane is very poorly documented with sequencing methods, which is surprising considering the importance of bread wheat as a crop. This situation probably results from sampling limitations for the root-sol interface and an increased focus on the root endosphere in recent years. In comparison with the rhizosphere, the rhizoplane displays a bacterial community that changes with wheat growth to a larger extent, with a decrease in *Proteobacteria* and an increase in *Actinobacteria* between the vegetative and ripening stages, as well as a decrease in *Bacteroidetes* associated with senescing roots compared to ripening stage (Donn et al., 2015). A lower abundance of *Acidobacteria* is observed at the rhizoplane in comparison with the rhizosphere (Donn et al., 2015).

#### Root Endosphere Microbiome

The bacterial community of the root endosphere of *T. aestivum*, in contrast with that of the rhizosphere, is dominated by *Actinobacteria* (Tkacz et al., 2020; Prudence et al., 2021). They represent about 40% of the total community based on culture-independent methods (Table 3), but <10% with culture-dependent methods (Germida and Siciliano, 2001; Bokati et al., 2016). They are followed by *Proteobacteria* (about 30%), and then *Bacteroidetes, Acidobacteria* and *Verrucomicrobia* (each at 5–10%). At family level, the *Streptomycetaceae* (*Actinobacteria*) dominates the endophytic community, followed by *Chitinophagaceae* (*Bacteroidetes*) and *Polyangiaceae* (*Proteobacteria*) (Prudence et al., 2021), whereas at genus level *Streptomyces, Microbispora*, *Micromonospora*, and *Nocardioides* (all in the *Actinobacteria* phylum) are prevalent (Coombs and Franco, 2003).

Root archaeal endophytes consist mainly of *Thaumarchaeota* (about 60% of the community) and *Euryarchaeota* (about 30%) (Table 3; Tkacz et al., 2020). It is a situation reminiscent of the one in the rhizosphere, but the abundance of *Thaumarchaeota* is lower in the root endosphere compared with the rhizosphere (Tkacz et al., 2020). The family *Nitrosphaeraceae* (*Thaumarchaeota*) also dominates in the root endosphere (about 75% of the community; Prudence et al., 2021). *Methanobacteriaceae* and *Methanocellaceae* are also present (about 10% of the community; Prudence et al., 2021).

For fungi, the predominance of *Ascomycota* in *T. aestivum* root endosphere (about 80%; Table 3) is documented with culture-independent and culture-dependent methods (Bokati et al., 2016; Özkurt et al., 2020). Basidiomycota represent <1% (Özkurt et al., 2020).

### Evolutionary History of Wheats and Microbiome Effects

#### Hybridization and Domestication

Inter-generic hybridizations and domestication events led to a range of wheat species with phenotypic differences between one another and with their wild progenitors (see above section). Wheat polyploidy is a trait thought to lead to slightly different bacterial community diversity in root and rhizosphere. Wipf and Coleman-Derr (2021) documented a higher abundance of *Actinobacteria* in roots of tetraploid and hexaploid species and a higher α-diversity in the rhizosphere, in comparison with diploids. Several studies investigated the impact of domestication on the microbial community of rhizosphere and root endosphere of wheat (Figure 2B; Kapulnik and Kushnir, 1991; Hetrick et al., 1992; Hassani et al., 2018; Özkurt et al., 2020; Tkacz et al., 2020; Wipf and Coleman-Derr, 2021). Comparison of *T. durum* and *T. aestivum* with *T. dicoccoides* and *A. tauschii* (Tkacz et al., 2020) evidenced the same bacterial phyla but not in the same proportions, depending on the wheat species and root compartment. In the rhizosphere, the abundance of *Verrucomicrobia* is lower and the abundance of *Actinobacteria* is higher for *T. dicoccoides* than the other wheats (Figure 3A; Tkacz et al., 2020). Wild varieties displayed higher bacterial α-diversity than domesticated ones when comparing diploid wheats (Wipf and Coleman-Derr, 2021). In the root endosphere, a higher proportion at the heading/flowering stage is found for *Bacteroidetes* in *T. dicoccoides*, *Chloroflexi* in *A. tauschii* and *Cyanobacteria* in *T. aestivum* compared with the other wheat species (Figure 3A; Tkacz et al., 2020). The root endosphere of seedlings displays a higher abundance of *Proteobacteria* and a lower abundance of *Firmicutes* for *T. dicoccoides* than for *T. aestivum* (Özkurt et al., 2020). Results with root samples pointed to higher α-diversity for wild polyploid wheats than domesticated polyploids, but shifts were of small magnitude (Wipf and Coleman-Derr, 2021). Higher stochasticity (e.g., priority effects) was found in *T. aestivum* than the wild species *T. dicoccoides* (Tkacz et al., 2020).

Archaea were studied (Tkacz et al., 2020), but sequencing targeted bacteria and archaea together, yielding limited numbers of sequences for archaea (<5%). This approach gave similar levels of *Thaumarcheota*, in the rhizosphere and the root endosphere, for *A. tauschii, T. dicoccoides, T. durum*, and *T. aestivum*.

For fungi, *A. tauschii* presented fewer *Zygomycota* in its rhizosphere than the other wheats did (Figure 3C; Tkacz et al., 2020). At genus level, fewer *Mortierella* (*Mucoromycota*) were found in *A. tauschii* and more *Verticillum* (*Ascomycota*) in *A. tauschii* and *T. dicoccoides* in comparison with *T. aestivum* (Tkacz et al., 2020). The less abundant fungal phylum in *T. aestivum* rhizosphere corresponded to the *Glomeromycota* (about 1%, Table 1), which were more abundant in the rhizosphere of *A. tauschii* (Tkacz et al., 2020). The selection of *Glomeromycota* and the plant response to these fungi are controlled by the D genome of *A. tauschii*, in comparison with genomes A (*T. urartu*) and B (*A. speltoides*) (Kapulnik and Kushnir, 1991; Hetrick et al., 1992; Zhu et al., 2001). The analysis of 32 *T. durum* genotypes indicated that *Glomeromycota* composition depended on plant genotype, and that certain *T. durum* genotypes associated strongly with *Paraglomus* and *Dominikia*, which were undetected in other genotypes (Ellouze et al., 2018). In the root endosphere, differences between *T. aestivum* and *T. dicoccoides* seedlings were evidenced, with a higher abundance (73 vs. 47%) of *Pleosporales* (*Ascomycota*) in *T. aestivum* than *T. dicoccoides* roots (Özkurt et al., 2020).

#### Post-domestication Selection

The analysis of bacterial rhizosphere isolates showed a higher diversity with a landrace compared with two cultivars of *T. aestivum*, with the genera *Aureobacter* and *Salmonella* found only in the landrace (Germida and Siciliano, 2001). With culture-independent methods, landraces presented in the rhizosphere a higher abundance of *Bacteroidetes* and a lower abundance of *Actinobacteria* in comparison with modern *T. aestivum* cultivars (Rossmann et al., 2020). Landraces also displayed a core microbiome with more bacterial genera that were specific, i.e., found only with landraces. In the rhizosphere, the fungal genus *Dominikia* (*Glomeromycota*) was detected with 80% of *T. durum* landraces but only 60% of modern genotypes (Ellouze et al., 2018). The endophytic fungal community of *T. durum* landrace Perciasacchi (winter type) is dominated by *Ascomycota* (with *Alternaria* and *Gibberella*) and that of *T. durum* landrace Tumminia (spring type) by *Basidiomycota* (and particularly *Sporobolomyces* and *Puccinia*) (Casini et al., 2019), but these landraces were not compared with durum wheat cultivars. When comparing root and rhizosphere microorganisms associated with landraces and modern cultivars of *T. aestivum*, old accessions were enriched in *Acidobacteria* and *Actinobacteria*, and modern cultivars in *Verrumicrobia* and *Firmicutes* (Kinnunen-Grubb et al., 2020). Landraces are genetically more heterogeneous than cultivars, including for traits that influence root-microorganism interactions, e.g., root system size and root exudation (Waines and Ehdaie, 2007; Haichar et al., 2008; Matthews et al., 2019; Iannucci et al., 2021), and thus at the scale of a field they are likely to select a wider range of soil microorganisms overall. At the scale of individual plants, however, the microbiota of both old and ancient bread wheats followed a neutral assembly model, and the root microbiome displayed higher stochasticity (i.e., less deterministic selection) with modern *T. aestivum* cultivars than with landraces (Kinnunen-Grubb et al., 2020).

The Green Revolution resulted in the selection of semi-dwarf cultivars. In comparison with older, tall cultivars, the rhizosphere bacterial community of semi-dwarf bread wheat cultivars displays lower levels of *Actinobacteria* (1.4 vs. 13%), *Bacteroidetes* (5.0 vs. 16%) and *Proteobacteria* (8.6 vs. 29%) and higher levels of *Verrucomicrobia* (7.9 vs. 2.9%), *Planctomycetes* (2.1 vs. 0.7%) and *Acidobacteria* (2.9 vs. 1.4%) (Kavamura et al., 2020). The latter phyla are typically well present in bulk soil, which suggests a lower selection intensity by semi-dwarf than tall cultivars (Kavamura et al., 2020). When considering rhizosphere selection of individual strains, the comparison of 192 *T. aestivum* varieties evidenced that old varieties had a higher ability than modern cultivars to recruit the bacterium *Pseudomonas ogarae* (ex-*fluorescens* ex-*kilonensis*) F113 (Valente et al., 2020). Root systems of mid and later-generation semi-dwarf wheats are smaller than those of early Green-Revolution wheats (Waines and Ehdaie, 2007), whereas the amount of simple sugars released by roots of modern wheats is higher (Shaposhnikov et al., 2016), probably due to less stringent control of sugar exudation (Pérez-Jaramillo et al., 2018). Much remains to be done to understand differences in microbial community between old and modern cultivars, notably for archaea and fungi (not considered so far).

Among modern bread wheat cultivars, differences in bacterial selection can be significant, for certain phyla (Figure 3A; Mahoney et al., 2017). The abundance of *Actinobacteria, Firmicutes* and *Cyanobacteria* in the rhizosphere of cultivar Madsen is lower than for cultivar Paragon (8 vs. 19%, 0.2 vs. 20%, and 0.5 vs. 2.8%, respectively; Figure 3A). Such differences can also be evidenced at lower taxonomic levels, and root colonization levels by *P. ogarae* F113 varied between modern *T. aestivum* cultivars (Valente et al., 2020). Different *T. aestivum* cultivars can present dissimilar abundance levels of *Thaumarchaeota* and *Euryarchaeota* (Figure 3B), but data scarcity does not enable to conclude on archaeal ecology (Fan et al., 2018; Tkacz et al., 2020; Prudence et al., 2021). With fungi, higher rhizosphere levels were found for *Basidiomycota* (>15% vs. <10%) in cultivars Paragon, SST88 and Eland (Gqozo et al., 2020; Tkacz et al., 2020) and for *Chytridiomycota* (about 20% vs. <10%) in cultivar Kariega (Gqozo et al., 2020; Figure 3C). Among 94 *T. aestivum* genotypes, variations in mycorrhizal colonization were observed following inoculation with *Rhizophagus* and *Claroideoglomus* species, which could vary depending on old vs. recent cultivars (Lehnert et al., 2017). The abundance of *Paraglomus* (*Glomeromycota*) depends on the modern *T. durum* cultivar in the rhizosphere but not in the root endosphere (Ellouze et al., 2018).

### Microbiome of Wheats vs. Other *Poaceae* and Non-*Poaceae*

#### Wheats vs. Other *Poaceae*

Compared with other *Poaceae*, Triticea members show some specificity in the level of certain phyla in the rhizosphere or root endosphere. Using selected publications (Supplementary Table 1), we found trends for (i) a higher abundance of *Cyanobacteria* and *Glomeromycota* and a lower abundance of *Firmicutes* in the rhizosphere, and (ii) a higher abundance of *Chloroflexi* in the root endosphere (Figures 3A,C). Differences between rhizobacterial communities increase with the phylogenetic distance between *Poaceae* (Bouffaud et al., 2016), but this is not apparent when considering phyla abundance. For example, *Verrucomicrobia* are found at the same level of magnitude in the rhizospheres of millet, rice and wheat (Figure 3A; Shi et al., 2019), even though the former two are distant from the Triticeae tribe. Moreover, rice (the closest to wheats in Figure 3C) displays a higher abundance of *Chytridiomycota* in the rhizosphere than Triticeae, whereas maize (although more distant) exhibits rhizosphere levels of *Chytridiomycota* closer to those of the Triticeae. This is also the case for the root endosphere, as the abundance of *Acidobacteria* in Triticeae is higher than in barley and oat but similar to levels in rice, sorghum and maize, which are comparatively more distant from wheats (Figure 3A). Nevertheless, such a variability between wheats and *Poaceae* needs to be considered in light of the high variability that exists between the different Triticeae species, and even between different *T. aestivum* cultivars.

#### Wheats vs. Non-*Poaceae*

Differences in bacterial community composition at phylum level can be found between Triticeae and non-*Poaceae*. This includes a lower abundance in rhizosphere and root endosphere of *Bacteroidetes* (for poplar) and *Chloroflexi* (for poplar and arabidopsis) compared with the Triticeae. A higher abundance of *Thaumarchaeota* (in rhizosphere and root endosphere) and *Nitrospirae* (in rhizosphere) is observed for tomato compared with *T. aestivum* (Figure 3B). In the rhizosphere, *Glomeromycota* are more abundant with Triticeae members than with tomato, bean, soybean and poplar (Figure 3C). However, significant microbiota similarities may also be observed when considering Triticeae and *non-Poaceae* plants mentioned in Figure 3. For instance, *Proteobacteria* and *Ascomycota* dominate the rhizobacterial community of both wheats and dicotyledons except for two varieties of *Arabidopsis thaliana* (Bulgarelli et al., 2012).

The comparison between Triticeae and non-*Poaceae* also reveals unexpected features, as certain differences do not coincide with the divide between these two groups. Barley (the closest to wheat in Figure 3A), displays surprisingly a low abundance of *Acidobacteria* in comparison with the Triticeae, the other *Poaceae* and also the non-*Poaceae*. The *Thaumarchaeota* dominate the rhizospheres of wheat and tomato, but not maize (Figure 3B). The rhizosphere and root endosphere of dicotyledons and certain *T. aestivum* are poorly colonized by *Zygomycota*, unlike for rice, other *Triticum* species and *A. tauschii.* In fact, extensive variability exists within the Triticeae, including between *T. aestivum* cultivars, and it is not necessarily lower than the variability between Triticeae and *non-Poaceae* observed in Figure 3.

## Functional Diversity of the Wheat Microbiome

### Functional Network of the Wheat Root Microbiome

Microorganisms play key roles in the biogeochemical cycles of carbon, nitrogen, sulfur, etc. (Philippot et al., 2013; Louca et al., 2016; Figure 1B). In root environments, the plant is the major provider of organic C and stimulate microorganisms, leading to the synthesis of various microbial metabolites, many of them with feed-back effects on the plant (Raaijmakers et al., 2009; Compant et al., 2010; Vacheron et al., 2013). The range of possible interactions between microorganisms and plant host is very broad (Figure 1B), from parasitism and competition to commensalism and mutualism (Lambers et al., 2009; Newton et al., 2010). Root-associated microorganisms also interact with one another, which modulates their own interactions with the plant (Raaijmakers et al., 2009; Huang et al., 2012). Due to the importance of root metabolism and rhizodeposition, it can be expected that exudate differences between wheat genotypes have the potential to materialize in significant differences in the implementation of biogeochemical cycles and biotic interactions in the root zone, but this possibility remains poorly documented.

Microbial functioning involves a complex network of elementary transformations (e.g., the conversion of N_2_ into NH_3_) or interactions (e.g., the inhibition mediated by a given antibiotic), each corresponding to a particular function. Each individual microorganism is endowed with many of these functions, and typically each function is common to different strains and species, leading to functional redundancy in the microbial community (Louca et al., 2016). All microorganisms participating to the same function form a functional group, and therefore each organism is likely to belong to several functional groups (Louca et al., 2016). For root-associated microorganisms, the context of the holobiont adds a supplementary dimension when considering microbial functioning (Vandenkoornhuyse et al., 2015; Lemanceau et al., 2017; Hassani et al., 2020). Whereas taxonomic variation within individual functional groups does not seem to fluctuate extensively with soil and other environmental conditions, the functional potential of the microbiota is thought to be strongly linked to environmental conditions (soil physico-chemistry, plant genotype, and growth stage) (Louca et al., 2016; Lemanceau et al., 2017; Guo et al., 2018). Accordingly, similar environments should promote similar microbial functional communities, while allowing for taxonomic variation inside an individual functional group.

### Global Functioning of the Wheat Microbiota

The emergence of metagenomics has made it possible to glimpse the global functional and metabolic capacities of a microbiota. The assessment of the rhizosphere metagenome of *T. durum* showed an overrepresentation of two categories of microbial functions (Ofek-Lalzar et al., 2016; Ofaim et al., 2017). A first category corresponded to basic metabolism, important for root colonization (Santi et al., 2013; Vacheron et al., 2013), such as chemotaxis, lipopolysaccharide metabolism, nitrogen metabolism, pentose and glucoronate interconversions, starch, and sucrose metabolism. The second category was related to secondary metabolism, e.g., anthocyanin production or xenobiotic metabolism (Ofaim et al., 2017; Lu et al., 2018). Whether metagenome differences occur between wheat species or lines of individual wheat species remains to be determined. Despite methodological limitations, functional metagenome predictions from metabarcoding-based OTU datasets did suggest microbial differences between wheat lines (Mahoney et al., 2017). These predictions differed also according to *T. aestivum* growth stage (Kavamura et al., 2018). Energy metabolism dominates in the rhizosphere microbiota during early wheat development, vs. degradation of complex organic compounds with older, photosynthetically active plants. A similar rhizosphere acclimatization was found with oat (Nuccio et al., 2020). Metatranscriptomic analysis of the rhizosphere of one *T. aestivum* genotype revealed metabolic capabilities for rhizosphere colonization, including cellulose degradation and methylotrophy (Turner et al., 2013).

Metagenomic or metatranscriptomic studies have been useful to describe global metabolic activity in root environments, but they have not been implemented yet to compare different wheat species or lines. Therefore, it remains difficult to assess the impact of intra-species variation, domestication and hybridization of wheats on global microbial functioning in the root zone, and this is a topic in strong need of research attention.

### Microbial Interactions in the Wheat Root and Rhizosphere

Rhizosphere microorganisms develop deleterious, beneficial or neutral interactions with one another (Figure 1B). The extent of these interactions, and the density and complexity of the resulting interaction network depend on taxa richness, microbial abundance and activity levels (Finlay et al., 1997; Torsvik and Øvreås, 2002; Fuhrman, 2009). Wheat root system architecture and rhizodeposition traits determine the root surface that can be colonized and the amount of root exudates. Since these characteristics were affected by domestication and subsequent crop selection (Waines and Ehdaie, 2007; Shaposhnikov et al., 2016; Iannucci et al., 2021), wild wheats, landraces and modern cultivars probably display different patterns of microbial colonization and of microbial interactions in their roots and rhizosphere. Microbial interactions and competition can also be modulated by predators (nematodes and protozoa), which thrive to different extents in the rhizospheres of oat, pea and wheat (Turner et al., 2013), and perhaps also in the rhizosphere of different wheat genotypes. Microbiota network analysis of *T. aestivum* rhizosphere revealed the co-occurrences of cercozoa (protozoa), bacterial and fungal taxa, reminiscent of a predator-prey system (Rossmann et al., 2020). Co-occurrence levels were higher and trophic networks more entangled with landraces than modern cultivars, which suggests a higher level of microbial interactions in the rhizosphere of landraces.

Pathogens infecting wheat roots cause significant damage, including the take-all fungus *Gaeumannomyces graminis* var. *tritici* (Wilkinson et al., 1985), *Rhizoctonia* spp. causing root rot or damping-off (Ingram and Cook, 1990), and *Pythium* species leading to root rot (Wiese, 1987; Ingram and Cook, 1990). Differences in sensitivity to root pathogens exist according to wheat genotype. *A. speltoides* and *T. durum* are more sensitive than *T. monococcum* to *G. graminis* var. *tritici* (McMillan et al., 2014), whereas inter-cultivar variability of resistance to this pathogen is high within *T. aestivum* (Golizadeh et al., 2017). *S*usceptibility to *Rhizoctonia* was similar for *T. monococcum* and *T. durum*, as well as certain *T. aestivum* cultivars, whereas other cultivars of *T. aestivum* were more sensitive (Oros et al., 2013). Differences in tolerance to *G. graminis* (Golizadeh et al., 2017), root-infecting *Fusarium graminearum* (Wang et al., 2018), foot, crown and root rot-causing *Fusarium culmorum* (Erginbaş Orakcı et al., 2018) and *Pythium* (Higginbotham et al., 2004) occurred among *T. aestivum* cultivars. Wheat is also affected by parasitic nematodes, with cultivar-level differences in susceptibility of *T. aestivum* to cereal cyst nematodes *Heterodera* spp. (Cui et al., 2016) and root-lesion nematode *Pratylenchus curvicauda* (Begum et al., 2020). Apart from differences in disease sensitivity, modern *T. aestivum* cultivars tend to be more colonized by *Fusarium*, *Neoascochyta* and *Microdochium* root pathogens compared with landraces (Kinnunen-Grubb et al., 2020).

Wheat cultivars might rely on specific microbial populations for phytoprotection, which may entail pathogen inhibition (*via* competition or antagonism) or systemic induction of plant defense pathways. Fluorescent *Pseudomonas* inhibit *Pythium, Rhizoctonia* and *G. graminis* (Weller and Cook, 1986; Mavrodi et al., 2013), using 2,4-diacetylphloroglucinol (DAPG), hydrogen cyanide (HCN), or phenazines (Keel et al., 1992; Kwak and Weller, 2013; Mavrodi et al., 2013; Imperiali et al., 2017). The diversity of bacterial populations producing these compounds and rhizosphere expression of the corresponding genes depend on plant host genotype (Latz et al., 2015). DAPG and HCN-producing microorganisms can be studied *via* the marker genes *phlD a*nd *hcnABC*, respectively (Supplementary Table 2), and DAPG-producing microorganisms associated with different wheat genotypes have been well-studied in comparison with the case of other antagonistic compounds (Table 4). Modern cultivars of *T. aestivum* differentially select for and benefit from DAPG-producing *Pseudomonas* species in resident soil populations (Berg et al., 2002; Mazzola et al., 2004; Meyer et al., 2010). Cultivar differences were evidenced in the interaction with DAPG-producing *P. brassicacearum* in soil suppressive to take-all (Yang et al., 2018). Suppression of *Rhizoctonia* root rot and take-all is cultivar-dependent, through enhanced recruitment of specific *Pseudomonas* populations by cultivars less affected by disease (Mazzola et al., 2004; Yang et al., 2018). Phytopathogens may also be inhibited by other saprophytic microorganisms, including bacteria and fungi (Barnett et al., 2017), and for the latter the ability to colonize wheat roots can depend on the cultivar (Osborne et al., 2018). In addition, Arbuscular Mycorrhizal (AM) fungi (division *Glomeromycota*) also inhibit wheat pathogens *via* competition (Ganugi et al., 2019) or production of cellulases and chitinases, which may affect pathogen cell wall and provide wheat protection (Pérez-de-Luque et al., 2017), but the significance of wheat genotypes is not documented. Induced resistance is poorly documented in monocots, including wheat (Balmer et al., 2013). Induced Systemic Resistance (ISR), which involves jasmonate and ethylene signaling, is triggered in wheat by certain *Pseudomonas* strains (Balmer et al., 2013). Similarly, saprophytic fungi from *Aspergillus*, *Penicillium* and *Trichoderma* genera and protecting against Rhizoctonia wilt trigger ISR in wheat (El-Maraghy et al., 2020). Mycorrhizae also induce systemic plant resistance, termed Mycorrhiza-Induced Resistance (MIR) (Mustafa et al., 2016), which is reminiscent of ISR (Balmer et al., 2013) but displays also features of Systemic Acquired Resistance (SAR), especially the priming of salicylic acid-dependent genes (Pérez-de-Luque et al., 2017). Thus, MIR by the AM fungus *Funneliformis mosseae* upregulated several defense genes in wheat, and protected wheat from the powdery mildew pathogen *Blumeria graminis* f. sp. *tritici* (Mustafa et al., 2016). In addition, *Bacillus velezensis* CC09 stimulated SAR pathways, inducing *PR1* genes and enhancing lignin accumulation, and protected wheat from take-all (Kang et al., 2018). Little has been done to compare induced resistance in different wheat genotypes (Table 4). The two cultivars studied in Pérez-de-Luque et al. (2017) displayed different levels of systemic priming for chitosan-induced callose after co-inoculation with *Pseudomonas putida* and *Rhizophagus irregularis.* One of the two cultivars showed higher level of callose deposition after co-inoculation than inoculation of *P. putida* or *R. irregularis* alone, suggesting additive or synergistic effects in some but not all genotypes, probably linked to cultivar differences in the signaling pathway leading to systemic immune priming (Haichar et al., 2016). Root expression of defense gene homologs induced by *P. brassicacearum* Q8r1-96 differed between the three wheat cultivars analyzed (Okubara et al., 2010). Since wheat species and varieties differ in their abilities to recruit microbial taxa containing strains with induced resistance potential, especially AM fungi (Figure 2A), it raises the possibility that some varieties are more prone to be protected by ISR. If so, this might explain some of the differences in sensitivity to diseases observed among cultivars.

**TABLE 4:** Literature comparisons of root-associated microbial functional groups considering (i) wheat of different species, wild or domesticated, (ii) landraces, ancient, or modern varieties within wheat species, and (iii) different modern cultivars within wheat species.

Microbial function	Analysis of individual microorganisms				Analysis of functional groups			
	Microorganism studied	(i) Wheat evolution/domestication	(ii) Genotype categories within wheat species	(iii) Wheat cultivars	Methodology used	(i) Wheat evolution/domestication	(ii) Genotype categories within wheat species	(iii) Wheat cultivars
**Biotic interactions**								
DAPG synthesis	*Pseudomonas brassicacearum* Q8r1-96			Okubara and Bonsall, 2008; Yang et al., 2018				
	*Pseudomonas fluorescens* Q2-87							
	*Pseudomonas ogarae* F113		Valente et al., 2020	Valente et al., 2020				
					PCR-RFLP and sequence analysis of *Pseudomonas* isolates			Mazzola et al., 2004
Phenazine synthesis	*Pseudomonas chlororaphis*			Mahmoudi et al., 2019				
Synthesis of antimicrobial compound(s)					*in silico* prediction from *rrs* metabarcodes			Mahoney et al., 2017
Fungal inhibition					PCR-RFLP of *Pseudomonas* isolates			Gu and Mazzola, 2003
*Rhizoctonia* inhibition					Agar plate assays of *Pseudomonas* isolates			Mazzola and Gu, 2002
Induction of root defense	*Pseudomonas brassicacearum* Q8r1-96			Maketon et al., 2012				
	*Pseudomonas putida*			Pérez-de-Luque et al., 2017				
	*Rhizophagus irregularis*							
	*Pseudomonas brassicacearum* Q8r1-96			Okubara et al., 2010				
IAA synthesis	*Azotobacter chroococcum*			Narula et al., 2000				
					Salkowski method and sequence analysis			Venieraki et al., 2011
ACC deaminase activity					Absorbance quantification of α-ketobutyrate product			Stromberger et al., 2017
Yield promotion	*Azospirillum brasilense* Cd	Pagnani et al., 2020						
	*Gluconacetobacter diazotrophicus* Pal5							
	*Herbaspirillum seropedicae* Z67							
**Biogeochemical cycles**								
Malate production					*in silico* prediction from *rrs* metabarcodes			Mahoney et al., 2017
Degradation of organic compound					Biolog™ plate assays		Siciliano et al., 1998	
Cellulose decomposition					Counts on cellulose Congo Red medium			Zuo et al., 2014
Urease, catalase, sucrose, and dehydrogenase synthesis					Colorimetric assays of potential enzymatic activities			Zuo et al., 2014
Nitrogen metabolism					Metabarcoding (*rrs*) predicted gene functioning			Mahoney et al., 2017
N_2_ fixation					Counts on N-free Ashby’s medium	Zuo et al., 2014		
					Acetylene reduction assays and *nifH* sequence analysis			Venieraki et al., 2011
					qPCR (*nifH*)	Spor et al., 2020		Rilling et al., 2018
Nitrification					Counts on improved Stephenson’s medium			Zuo et al., 2014
					qPCR (*amoA*)	Spor et al., 2020		
Denitrification					Measurement of nitrate reductase potential activity			Gill et al., 2006
					qPCR (*nirK/nirS, nosZI/nosZII*)	Spor et al., 2020		
Sulfur metabolism					*in silico* prediction from *rrs* metabarcodes			Mahoney et al., 2017
Phosphorus metabolism					*in silico* prediction from *rrs* metabarcodes			Mahoney et al., 2017
Phosphate solubilization	*Azotobacter chroococcum*			Narula et al., 2000				

*These comparisons were carried out at the level of individual microorganisms (whereby one or several microorganisms was/were inoculated on wheat) or entire functional groups (i.e., taking into account most or all microorganisms potentially contributing to a given microbial function). RFLP, Restriction Fragment Length Polymorphism. List of references is available in Supplementary Material 1.*

Apart from plant protection, several microbial functional groups present in the rhizosphere are beneficial, by ensuring better plant growth (Vacheron et al., 2013; Lemanceau et al., 2017). However, information about their abundance and activity according to wheat genotype is scarce, even for the best described functional groups. Mineral nutrition of wheat can be promoted by different functional groups directly affecting nutrient bioavailability or stimulating plant development through microbial modulation of its hormonal balance (Finkel et al., 2017). Wheat growth can be promoted by microorganisms that produce phytohormones, such as Indole-3-Acetic Acid (IAA) (Spaepen et al., 2007), cytokinins and gibberellin, or secondary metabolites interfering with auxin, such as DAPG (Landa et al., 2003) and nitric oxide (NO) (Molina-Favero et al., 2008). Inoculation with different P-solubilizing, IAA-producing strains of *Azotobacter chroococcum* carried out on three cultivars of *T. aestivum* showing contrasted P responses resulted in enhanced N, P, K uptakes, probably as a consequence of phytohormone effects, but without difference between wheat genotypes (Narula et al., 2000). In contrast, cultivar differences were found following *Azospirillum* inoculation, when considering phenotypic traits such as root system architecture or plant height, especially during early growth phases, but without an effect on grain yield (Kazi et al., 2016). Using the Opata × synthetic mapping population, one QTL region on chromosome 1A was identified for *Azospirillum* adhesion to wheat roots (Díaz De León et al., 2015). DAPG, at low concentration, induces the plant’s auxinic pathways, which stimulates root exudation and branching (Brazelton et al., 2008; Combes-Meynet et al., 2010). Application of wheat root exudates to a soil modified the composition of the DAPG-producing community in a cultivar-specific manner (Gu and Mazzola, 2003). The activity of DAPG-producing *Pseudomonas* in the rhizosphere and DAPG accumulation on the rhizoplane of *T. aestivum* is influenced by specific cultivar-bacterial strain associations (Bergsma-Vlami et al., 2005; Okubara et al., 2010). Variability is also observed between old and modern cultivars, as colonization by *P. ogarae* F113 and expression of *phl* genes (coding for DAPG production) are higher for ancient genotypes of *T. aestivum* (Valente et al., 2020). Not much data is available on the effect of wheat genotype on the abundance or activity of other phytohormone-producing microorganisms (Table 4). 1-aminocyclopropane-1-carboxylate (ACC) is the precursor of ethylene in plants and more ACC is produced in case of stress, which may have deleterious effects on plant growth (Glick, 2014). ACC deaminase activity, which catalyzes the cleavage of ACC into ammonium and alpha-ketobutyrate (Glick, 2005, 2014), is found in many microorganisms living in the rhizosphere (Bouffaud et al., 2018). This activity can improve the growth and yield of *T. aestivum* under salt stress and drought conditions, acting as an ACC sink that lowers ethylene level without stopping stress-induced reactions (Zahir et al., 2009; Shakir et al., 2012; Hassan et al., 2014). The abundance of microorganisms with ACC deaminase activity in the rhizosphere varies with plant genotype (Bouffaud et al., 2018), and the abundance, composition and activity of the corresponding functional group depends also on *T. aestivum* cultivar (Stromberger et al., 2017). Under drought or well-watered conditions, the response of *T. aestivum* to inoculation with ACC deaminase-producing bacteria is genotype dependent, as some cultivars showed higher root length while others had increased above-ground biomass (Salem et al., 2018). The ability of these bacteria to promote drought resistance may depend on wheat genotype (Stromberger et al., 2017). The significance of ACC deaminase activity has not been studied for wheat species other than *T. aestivum* (Figure 2A). *Pseudomonas* producing phenazine (usually studied for its antimicrobial properties) are thought to be involved in drought resistance as well. Indeed, when inoculated, their presence on roots (at population levels that are wheat cultivar dependent; Mahmoudi et al., 2019) leads to added protection of seedlings against drought in cultivars that are genetically drought resistant (Mahmoudi et al., 2019). Moreover, *Pseudomonas* producing phenazine were shown to be abundant in non-irrigated soil (Mavrodi et al., 2012, 2013). Furthermore, resistance to drought (along with other abiotic stress like salinity or metals; Seguel et al., 2016; Aguilera et al., 2018; Ganugi et al., 2019) may be conferred by AM fungi following the induction of particular metabolomic responses in wheat roots (Bernardo et al., 2019). This protection varies with *T. aestivum* cultivars and QTLs have been identified, especially on chromosomes 3D and 7D (Lehnert et al., 2017). More generally, genome-wide association studies for the establishment of AM symbiosis have highlighted QTL regions on chromosomes 3A, 4A, and 7A in *T. aestivum* inoculated with *Rhizophagus intraradices*, *Claroideoglomus claroideum* and *Claroideoglomus etunicatum* (Lehnert et al., 2017) and on chromosomes 1A, 2B, 5A, 6A, 7A, and 7B for *T. durum* when inoculated individually with *Funneliformis mosseae* or *Rhizoglomus irregulare* (De Vita et al., 2018). Depending on the species (*T. aestivum* or *T. durum*), changes in rhizosphere microbiota traits due to drought will differ, suggesting that different wheat genotypes recruit their own specific microbiota to help alleviate abiotic stresses (Azarbad et al., 2018, 2020).

### Biogeochemical Cycles in the Wheat Root and Rhizosphere

The rhizosphere is characterized by the release of organic exudates and other rhizodeposits, and the uptake of mineral nutrients by roots (Figure 1B). The biogeochemical cycles of carbon, nitrogen and phosphorus are well-understood, and several primers are available to target microbial markers associated with these cycles (Supplementary Table 2). However, microbial activities involved in other important cycles such as those of potassium, sulfur or iron are less described in the wheat root and rhizosphere (Narula et al., 2000; Sheng and He, 2011; Jacoby et al., 2017; Kumar et al., 2018).

#### Carbon

Carbon efflux from the root is important (Kuzyakov and Cheng, 2001) and wheat root-derived CO_2_ represents between 25 and 50% of the total CO_2_ efflux from soil (Kuzyakov and Cheng, 2001). Most microbial functions related to carbon in the rhizosphere are linked to degradation of organic exudates (Derrien et al., 2004; Haichar et al., 2016). The rhizosphere priming effect (i.e., the increase of microbial activity following exudation) intensifies decomposition and mineralization (Cheng et al., 2003), and is an important cause of carbon loss from the rhizosphere.

Bacteria and fungi are primary decomposers, releasing extracellular hydrolytic enzymes to catalyze decomposition of organic matter (Berg and McClaugherty, 2008). Actinobacteria, found in the rhizosphere of *T. aestivum* (Table 1) include different species involved in decomposition and humus formation (Wang C. et al., 2016; Bao et al., 2021). In the rhizosphere, the potential activity of microbial enzymes (mostly cellulases) associated with carbohydrate degradation differed according to *T. aestivum* cultivar (Table 4; Zuo et al., 2014). Moreover, endophytic microorganisms isolated from roots of ancient *T. durum* cultivars differed in their ability to degrade organic compounds compared with those from more recent cultivars (Siciliano et al., 1998). Indeed, microorganisms isolated from recent cultivar CDC Teal degraded carboxylic acids at a higher rate, whereas polymers and amino acids were degraded at a higher rate by microorganisms isolated from ancient cultivars Red Fife and PI 167549.

#### Nitrogen

Diazotrophs are well-represented in the plant rhizosphere, where they find sufficient energy resources for the costly functioning of the dinitrogenase that fixes N_2_ into assimilable NH_3_ (Herridge et al., 2008). N_2_ fixation is the best studied activity when comparing different wheat genotypes (Table 4). The ability of N_2_-fixing *Azospirillum* to colonize roots depends on *T. aestivum* cultivar, as the bacteria were detected either in the root tissues and intercellular spaces or only at the root surface (Schloter and Hartmann, 1998). *Cyanobacteria* of the genera *Nostoc* (Gantar et al., 1993) and *Azospirillum brasilense* FP2 fix N_2_ when colonizing *T. aestivum* roots (Van Dommelen et al., 2009; Camilios-Neto et al., 2014). Free-living nitrogen-fixing prokaryotes contribute to nitrogen requirement of wheat (Dellagi et al., 2020), up to 76 and 32% for shoots and roots, respectively (Majeed et al., 2015). In addition, higher yields were observed for *T. aestivum* inoculated with engineered strains able to fix nitrogen constitutively (Fox et al., 2016). The diazotroph community varies in size and activity with plant species (Perin et al., 2006; Mao et al., 2013; Bouffaud et al., 2016) and cultivars of *T. aestivum*, with a higher number of rhizosphere diazotrophs for Xiaoyan than for other *T. aestivum* cultivars (Mahoney et al., 2017).

Even though N is often limiting for plant growth, wheat roots may release nitrogen in the form of NH_3_/NH_4_^+^ in the rhizosphere (26 mg for the entire growing season, 18% of the total N yield of the plant; Janzen, 1990). Within the wheat rhizosphere, NH_3_ is oxidized into NO_2_^–^ and NO_3_^–^ by aerobic nitrifiers. Ammonium oxidizers include ammonium-oxidizing bacteria (AOB) and ammonium-oxidizing archaea (AOA; Chen et al., 2011). Wheat domestication and selection had an effect on the interaction with nitrifiers, as they are less abundant in the rhizosphere of modern *T. durum* cultivars compared with *T. dicoccoides* and *T. dicoccon* (Spor et al., 2020). Differences in abundance of nitrifying bacteria also exist between *T. aestivum* cultivars, with higher rhizosphere numbers for cultivar Xiaoyan than the others (Mahoney et al., 2017). In addition, some *T. aestivum* landraces can inhibit nitrification in their rhizosphere (O’Sullivan et al., 2016).

Denitrifying bacteria harboring NO_2_^–^ reductase genes *nirK*/*nirS* (Chen et al., 2011) reduce NO_2_^–^ into NO. In the *T. aestivum* rhizosphere, denitrification is stimulated by root exudates (as denitrifiers are heterotrophs) (Wollersheim et al., 1987) and soil waterlogging (as it results in anoxia) (Hamonts et al., 2013). Thus, *T. aestivum* modulates denitrification activity and influences the composition of the denitrifying community (Achouak et al., 2019). Among modern *T. aestivum* cultivars, differences in rhizosphere denitrification activity and N_2_O emissions are significant (Hayashi et al., 2015), including for cultivars that can even inhibit nitrate reductase activity (Gill et al., 2006).

#### Phosphorus

Phosphorus is mostly present as insoluble phosphate or organic forms in the soils. Microorganisms can degrade P-containing organic matter *via* phosphatases, thereby mineralizing phosphorus and making it potentially available for plants. Some bacteria also act as Phosphate-Solubilizing Bacteria (PSB), thanks to the production of organic acids, protons, IAA (Gyaneshwar et al., 2002). PSB have been isolated from the rhizosphere of *T. aestivum*, e.g., *Streptomyces* spp. (Jog et al., 2014), *Pseudomonas* sp. BR2 (Babana et al., 2013) and *Bacillus* sp. (Majeed et al., 2015), and the rhizosphere of *T. durum* (Cherchali et al., 2019; Di Benedetto et al., 2019). The number of culturable PSB varies with plant species (Kundu et al., 2009). Since PSB are heterotrophic, their abundance in the rhizosphere of *T. aestivum* is influenced by organic matter content (Abderrazak et al., 2017), and differences between wheat cultivars might be expected since each may exude differently (Waines and Ehdaie, 2007; Iannucci et al., 2021). Wheat cultivars differed in the abundance of microbiota sequences linked to phosphate metabolism (Mahoney et al., 2017). Inoculation with a PSB from *Azotobacter chroococcum* in the rhizosphere of three cultivars of *T. aestivum* increased the number of grains per spike, straw yield, and root biomass, but without significant difference between cultivars (Narula et al., 2000). This is the only study dealing with P solubilization activity in different wheat cultivars (Table 4).

In addition to bacteria, AM fungi associated to wheat can mineralize organic phosphorus. The symbiotic network formed by mycorrhizal fungi with plant roots increases the volume of soil exploited for nutrients, providing the plant with P sources while the fungus acquires organic carbon from the plant (Hamel et al., 2004; Li et al., 2006; Pellegrino et al., 2015). The importance of AM fungi in wheat phosphorus feeding depends on the combination of plant and fungal genotypes (Hamel et al., 2004; Pérez-de-Luque et al., 2017). Indeed, the abundance of AM fungi differs between *T. durum* varieties, with a higher abundance associated with landraces than with modern cultivars (Ellouze et al., 2018). Similarly, wild wheats of genome A (*T. urartu*) and B (*A. speltoides*) showed more mycorrhizal dependence (i.e., the degree of plant growth and nutrition obtained with the help of AM fungi) than wild wheat of the D genome (*A. tauschii*) (Hetrick et al., 1992). Thus, the response to AM fungi in hexaploid wheat is probably controlled by the D genome (Kapulnik and Kushnir, 1991; Hetrick et al., 1992). Mycorrhizal dependence was lower in modern cultivars in comparison to old cultivars of *T. aestivum* (Zhu et al., 2001). Modern wheat crops seem to select their partner less (Hetrick et al., 1992; Zhu et al., 2001) and mycorrhizal dependence has been reported to decrease with wheat domestication (domesticated *T. durum* vs. wild *T. dicoccoides*; Martín-Robles et al., 2018). Indeed, in agroecosystems where different fertilization regimes are applied, modern cultivars probably rely less on microorganisms for their nutrition since they are well-provided with fertilizers (Duhamel and Vandenkoornhuyse, 2013).

## Conclusion

Wheat, one of the three most important crops in the world, has undergone a particularly complex evolutionary history involving several inter-genus crosses, genomic hybridizations and domestication events, which resulted in the formation of several wheat species able to grow in contrasted climates and cultivated (mostly *T. aestivum* and *T. durum*) for various feed and food purposes. The implications of this very particular evolutionary history on the recruitment and functioning of root and rhizosphere microbiomes are poorly understood.

Most studies have focused on the taxonomic features of these microbiomes, describing the abundance and diversity of microorganisms associated with wheat species. The most dominant bacterial phylum corresponds to *Proteobacteria* in the rhizosphere (as for other plant taxa) and *Actinobacteria* in the root endosphere (but *Proteobacteria* for other *Poaceae* and for non-*Poaceae* taxa). In both compartments, the archaeal and fungal communities are dominated by, respectively, *Thaumarchaeota* (as for other plant taxa) and *Ascomycota* (as for the other plant taxa investigated except poplar, where *Basidiomycota* dominate).

Domestication, selection and modern breeding created wheats selecting different root and rhizosphere communities in comparison with those of their wild or ancient relatives. Evidence for the importance of genomic hybridizations relates especially to the case of *Glomeromycota*, whose mycorrhizal association is controlled by D genome factors. Otherwise, the main differences in root and rhizosphere microorganisms seem to stem from post-domestication selection, based on the information available so far. Indeed, landraces are associated with a larger microbial diversity, which probably results from their higher plant-to-plant genetic heterogeneity, and their core microbiome presents certain bacterial families not found in modern cultivars. However, at the level of individual plants, strong microbial selection takes place in the rhizosphere of landraces, with higher taxa co-occurrence levels. This is attributed to a stronger selective pressure in the rhizosphere of pre-Green Revolution wheat compared to semi-dwarf varieties and modern cultivars of *T. aestivum*. Overall, the vast majority of analyses considering wheat genetic diversity have been restricted to the comparison of different cultivars of *T. aestivum*, showing microbial variability between them, to an extent not necessarily lower than that found between Triticeae and other *Poaceae* or *non-Poaceae.*

At the functional level, much less is documented on root-associated microorganisms in comparison with taxonomic data. Information is scarce on their functional traits, both for metagenomic and metatranscriptomic investigations targeting the entire microbial community and studies dealing with particular microbial functional groups. Differences in recruitment of disease-suppressive microorganisms are seen between cultivars of *T. aestivum*, contributing to differences in wheat health. The best-documented impact of domestication and post-domestication selection concerns the ability to interact and benefit from AM fungi, which decreases along the domestication/selection gradient.

Overall, it appears that wheat evolution has resulted into crop varieties with particular microbiome profiles, which probably rely less on their underground microbial partners for provision of growth resources and protection against diseases, and thus they are more dependent on human management. The development of omics tools targeting microbial functions in the rhizosphere is expected to provide new insights into the significance of wheat domestication and diversification for wheat-microorganisms interactions. It should also facilitate the design of novel breeding strategies integrating the contribution of root symbiotic partners for sustainable wheat farming.

## Author Contributions

All authors contributed to the writing of this review article and approved the submitted version.

## Conflict of Interest

The authors declare that the research was conducted in the absence of any commercial or financial relationships that could be construed as a potential conflict of interest.

## Publisher’s Note

All claims expressed in this article are solely those of the authors and do not necessarily represent those of their affiliated organizations, or those of the publisher, the editors and the reviewers. Any product that may be evaluated in this article, or claim that may be made by its manufacturer, is not guaranteed or endorsed by the publisher.

## Abbreviations

BP: Before Present
DAPG: 2,4-Diacetylphloroglucinol
AM: Arbuscular Mycorrhizal
ISR: Induced Systemic Resistance
MIR: Mycorrhiza-Induced Resistance
SAR: Systemic Acquired Resistance
QTL: Quantitative Trait Loci
IAA: Indole-3-Acetic Acid
ACC: 1-Aminocyclopropane-1-Carboxylate
PSB: Phosphate-Solubilizing Bacteria.

## References

[B1] Abderrazak R. Laila N. Jamal I. (2017). Occurrence of phosphate solubilizing bacteria in the rhizosphere of *Triticum aestivum* L. from Meknes, Morocco. *Am. J. Microbiol. Biotechnol.* 4 1–7.

[B2] Achouak W. Abrouk D. Guyonnet J. Barakat M. Ortet P. Simon L. (2019). Plant hosts control microbial denitrification activity. *FEMS Microbiol. Ecol.* 95:fiz021. 10.1093/femsec/fiz021 30726948

[B3] Aguilera P. Larsen J. Borie F. Berríos D. Tapia C. Cornejo P. (2018). New evidences on the contribution of arbuscular mycorrhizal fungi inducing Al tolerance in wheat. *Rhizosphere* 5 43–50. 10.1002/jsfa.10088 31612503

[B4] Ahlawat O. P. Tiwari R. Singh G. P. (2018). Metagenomics of wheat rhizosphere for abiotic stress management. *Wheat Barley Res.* 10 64–77.

[B5] Azarbad H. Constant P. Giard-Laliberté C. Bainard L. D. Yergeau E. (2018). Water stress history and wheat genotype modulate rhizosphere microbial response to drought. *Soil Biol. Biochem.* 126 228–236. 10.1016/j.soilbio.2018.08.017

[B6] Azarbad H. Tremblay J. Giard-Laliberté C. Bainard L. D. Yergeau E. (2020). Four decades of soil water stress history together with host genotype constrain the response of the wheat microbiome to soil moisture. *FEMS Microbiol. Ecol.* 96:fiaa098. 10.1093/femsec/fiaa098 32440671

[B7] Babana A. H. Dicko A. H. Maïga K. Traoré D. (2013). Characterization of rock phosphate-solubilizing microorganisms isolated from wheat (*Triticum aestivum* L.) rhizosphere in Mali. *J. Microbiol. Microbial Res.* 1 1–6.

[B8] Badri D. V. Vivanco J. M. (2009). Regulation and function of root exudates. *Plant Cell Environ.* 32 666–681. 10.1111/j.1365-3040.2008.01926.x 19143988

[B9] Balmer D. Planchamp C. Mauch-Mani B. (2013). On the move: induced resistance in monocots. *J. Exp. Bot.* 64 1249–1261. 10.1093/jxb/ers248 23028020

[B10] Bao Y. Dolfing J. Guo Z. Chen R. Wu M. Li Z. (2021). Important ecophysiological roles of non-dominant Actinobacteria in plant residue decomposition, especially in less fertile soils. *Microbiome* 9: 84. 10.1186/s40168-021-01032-x 33827695 PMC8028251

[B11] Barnett S. Zhao S. Ballard R. Franco C. (2017). Selection of microbes for control of Rhizoctonia root rot on wheat using a high throughput pathosystem. *BiolControl* 113 45–57.

[B12] Begum F. Jones M. G. K. Fosu-Nyarko J. (2020). Assessment of the pest status of *Pratylenchus curvicauda* and ultrastructural changes in roots of infected wheat and barley. *Plant Pathol* 69 1574–1588. 10.1111/ppa.13232

[B13] Berg B. McClaugherty C. (2008). “Decomposition of fine root and woody litter,” in *Plant Litter: Decomposition, Humus Formation, Carbon Sequestration*, eds Berg B. McClaugherty C. (Berlin: Springer), 193–209. 10.1007/978-3-540-74923-3_9

[B14] Berg G. Smalla K. (2009). Plant species and soil type cooperatively shape the structure and function of microbial communities in the rhizosphere. *FEMS Microbiol. Ecol* 68 1–13. 10.1111/j.1574-6941.2009.00654.x 19243436

[B15] Berg G. Roskot N. Steidle A. Eberl L. Zock A. Smalla K. (2002). Plant-dependent genotypic and phenotypic diversity of antagonistic rhizobacteria isolated from different *Verticillium* host plants. *Appl. Environ. Microbiol.* 68 3328–3338. 10.1128/AEM.68.7.3328-3338.2002 12089011 PMC126805

[B16] Bergsma-Vlami M. Prins M. E. Raaijmakers J. M. (2005). Influence of plant species on population dynamics, genotypic diversity and antibiotic production in the rhizosphere by indigenous *Pseudomonas* spp. *FEMS Microbiol. Ecol.* 52 59–69. 10.1016/j.femsec.2004.10.007 16329893

[B17] Bernardo L. Carletti P. Badeck F. W. Rizza F. Morcia C. Ghizzoni R. (2019). Metabolomic responses triggered by arbuscular mycorrhiza enhance tolerance to water stress in wheat cultivars. *Plant Physiol. Biochem.* 137 203–212. 10.1016/j.plaphy.2019.02.007 30802803

[B18] Bhattacharyya P. N. Jha D. K. (2012). Plant growth-promoting rhizobacteria (PGPR): emergence in agriculture. *World J. Microbiol. Biotechnol.* 28 1327–1350. 10.1007/s11274-011-0979-9 22805914

[B19] Bokati D. Herrera J. Poudel R. (2016). Soil influences colonization of root-associated fungal endophyte communities of maize, wheat, and their progenitors. *J. Mycol.* 2016:8062073.

[B20] Bonjean A. (2001). Histoire de la culture des céréales et en particulier de celle du blé tendre (*Triticum aestivum* L.). *Doss. Environ. INRA* 21 29–37.

[B21] Bonnin I. Bonneuil C. Goffaux R. Montalent P. Goldringer I. (2014). Explaining the decrease in the genetic diversity of wheat in France over the 20th century. *Agric. Ecosyst. Environ.* 195 183–192. 10.1007/s00122-005-2014-8 15887038

[B22] Bouffaud M. L. Renoud S. Dubost A. Moënne-Loccoz Y. Muller D. (2018). 1-Aminocyclopropane-1-carboxylate deaminase producers associated to maize and other *Poaceae* species. *Microbiome* 6:114. 10.1186/s40168-018-0503-7 29925415 PMC6011333

[B23] Bouffaud M. L. Renoud S. Moënne-Loccoz Y. Muller D. (2016). Is plant evolutionary history impacting recruitment of diazotrophs and *nifH* expression in the rhizosphere? *Sci. Rep.* 6:21690. 10.1038/srep21690 26902960 PMC4763242

[B24] Bouffaud M. Poirier M. Muller D. Moënne-Loccoz Y. (2014). Root microbiome relates to plant host evolution in maize and other Poaceae. *Environ. Microbiol.* 16 2804–2814. 10.1111/1462-2920.12442 24588973

[B25] Brancourt-Hulmel M. Doussinault G. Lecomte C. (2003). Genetic improvement of agronomic traits of winter wheat cultivars released in France from 1946 to 1992. *Crop Sci.* 43 37–45. 10.2135/cropsci2003.0037 34798789

[B26] Brazelton J. N. Pfeufer E. E. Sweat T. A. McSpadden Gardener B. B. Coenen C. (2008). 2,4-Diacetylphloroglucinol alters plant root development. *Mol. Plant-Microbe Interact.* 21 1349–1358. 10.1094/MPMI-21-10-1349 18785830

[B27] Brisson N. Gate P. Gouache D. Charmet G. Oury F. X. Huard F. (2010). Why are wheat yields stagnating in Europe? A comprehensive data analysis for France. *Field Crops Res.* 119 201–212.

[B28] Buée M. De Boer W. Martin F. van Overbeek L. Jurkevitch E. (2009). The rhizosphere zoo: an overview of plant-associated communities of microorganisms, including phages, bacteria, archaea, and fungi, and of some of their structuring factors. *Plant Soil* 321 189–212. 10.1007/s11104-009-9991-3

[B29] Bulgarelli D. Rott M. Schlaeppi K. Ver Loren van Themaat E. Ahmadinejad N. Assenza F. (2012). Revealing structure and assembly cues for *Arabidopsis* root-inhabiting bacterial microbiota. *Nature* 488 91–95. 10.1038/nature11336 22859207

[B30] Camilios-Neto D. Bonato P. Wassem R. Tadra-Sfeir M. Z. Brusamarello-Santos L. C. Valdameri G. (2014). Dual RNA-seq transcriptional analysis of wheat roots colonized by *Azospirillum brasilense* reveals up-regulation of nutrient acquisition and cell cycle genes. *BMC Genomics* 15:378. 10.1186/1471-2164-15-378 24886190 PMC4042000

[B31] Cantarel A. A. M. Allard V. Andrieu B. Barot S. Enjalbert J. Gervaix J. (2021). Plant functional trait variability and trait syndromes among wheat varieties: the footprint of artificial selection. *J. Exp. Bot.* 72 1166–1180. 10.1093/jxb/eraa491 33080022

[B32] Casini G. Yaseen T. Abdelfattah A. Santoro F. Varvaro L. Drago S. (2019). Endophytic fungal communities of ancient wheat varieties. *Phytopathol. Mediter.* 58 151–162.

[B33] Chaudhary S. Dheri G. S. Brar B. S. (2017). Long-term effects of NPK fertilizers and organic manures on carbon stabilization and management index under rice-wheat cropping system. *Soil Tillage Res.* 166 59–66. 10.1016/j.still.2016.10.005

[B34] Chen X. Zhang L. M. Shen J. P. Wei W. X. He J. Z. (2011). Abundance and community structure of ammonia-oxidizing archaea and bacteria in an acid paddy soil. *Biol. Fertil. Soils* 47 323–331.

[B35] Cheng W. Johnson D. W. Fu S. (2003). Rhizosphere effects on decomposition. *Soil Sci. Soc. Am. J.* 67 1418–1427. 10.2136/sssaj2003.1418

[B36] Cherchali A. Boukhelata N. Kaci Y. Abrous-Belbachir O. Djebbar R. (2019). Isolation and identification of a phosphate-solubilizing *Paenibacillus polymyxa* strain GOL 0202 from durum wheat (*Triticum durum* desf.) rhizosphere and its effect on some seedlings morphophysiological parameters. *Biocatal. Agric. Biotechnol.* 19:101.

[B37] Combes-Meynet E. Pothier J. F. Moënne-Loccoz Y. Prigent-Combaret C. (2010). The *Pseudomonas* secondary metabolite 2,4-diacetylphloroglucinol is a signal inducing rhizoplane expression of *Azospirillum* genes involved in plant-growth promotion. *Mol. Plant-Microbe Interact.* 24 271–284. 10.1094/MPMI-07-10-0148 21043573

[B38] Compant S. Clément C. Sessitsch A. (2010). Plant growth-promoting bacteria in the rhizo- and endosphere of plants: their role, colonization, mechanisms involved and prospects for utilization. *Soil Biol. Biochem.* 42 669–678. 10.1016/j.soilbio.2009.11.024

[B39] Coombs J. T. Franco C. M. M. (2003). Isolation and Identification of *Actinobacteria* from surface-sterilized wheat roots. *Appl. Environ. Microbiol.* 69 5603–5605. 10.1128/aem.69.9.5603-5608.2003 12957950 PMC194995

[B40] Cui L. Sun L. Gao X. Song W. Wang X. M. Li H. L. (2016). The impact of resistant and susceptible wheat cultivars on the multiplication of *Heterodera filipjevi* and *H. avenae* in parasite-infested soil. *Plant Pathol.* 65 1192–1199. 10.1111/ppa.12495

[B41] De Vita P. Avio L. Sbrana C. Laidò G. Marone D. Mastrangelo A. M. (2018). Genetic markers associated to arbuscular mycorrhizal colonization in durum wheat. *Sci. Rep.* 8:10. 10.1038/s41598-018-29020-6 30006562 PMC6045686

[B42] Dellagi A. Quillere I. Hirel B. (2020). Beneficial soil-borne bacteria and fungi: a promising way to improve plant nitrogen acquisition. *J. Exp. Bot.* 71 4469–4479. 10.1093/jxb/eraa112 32157312 PMC7475097

[B43] Derrien D. Marol C. Balesdent J. (2004). The dynamics of neutral sugars in the rhizosphere of wheat: an approach by 13C pulse-labelling and GC/C/IRMS. *Plant Soil* 267:243.

[B44] Di Benedetto N. A. Campaniello D. Bevilacqua A. Cataldi M. P. Sinigaglia M. Flagella Z. (2019). Isolation, screening, and characterization of plant-growth-promoting bacteria from durum wheat rhizosphere to improve N and P nutrient use efficiency. *Microorganisms* 7:541. 10.3390/microorganisms7110541 31717409 PMC6920805

[B45] Di Paola A. Caporaso L. Di Paola F. Bombelli A. Vasenev I. Nesterova O. V. (2018). The expansion of wheat thermal suitability of Russia in response to climate change. *Land Use Policy* 78 70–77.

[B46] Díaz De León J. L. Castellanos T. Ling J. Rojas-Hernández A. Röder M. S. (2015). Quantitative trait loci underlying the adhesion of *Azospirillum brasilense* cells to wheat roots. *Euphytica* 204 81–90. 10.1007/s10681-014-1334-7

[B47] Ding L. J. Cui H. L. Nie S. A. Long X. E. Duan G. L. Zhu Y. G. (2019). Microbiomes inhabiting rice roots and rhizosphere. *FEMS Microbiol. Ecol.* 95:fiz040. 10.1093/femsec/fiz040 30916760

[B48] Dong G. Wei M. Yang Y. Liu R. Wang J. Chen L. (2019). A brief history of wheat utilization in China. *Front Agr. Sci. Eng.* 6 288–295. 10.15302/J-FASE-2019266

[B49] Dong W. Liu E. Yan C. Tian J. Zhang H. Zhang Y. (2017). Impact of no tillage vs. conventional tillage on the soil bacterial community structure in a winter wheat cropping succession in northern China. *Eur. J. Soil Biol.* 80 35–42.

[B50] Donn S. Kirkegaard J. A. Perera G. Richardson A. E. Watt M. (2015). Evolution of bacterial communities in the wheat crop rhizosphere. *Environ. Microbiol.* 17 610–621. 10.1111/1462-2920.12452 24628845

[B51] Duhamel M. Vandenkoornhuyse P. (2013). Sustainable agriculture: possible trajectories from mutualistic symbiosis and plant neodomestication. *Trends Plant Sci.* 18 597–600. 10.1016/j.tplants.2013.08.010 24055138

[B52] Dvorak J. Luo M. C. Yang Z. L. Zhang H. B. (1998). The structure of the *Aegilops tauschii* genepool and the evolution of hexaploid wheat. *Theor. Appl. Genet.* 97 657–670. 10.1016/j.compbiolchem.2019.107144 31751884

[B53] Dwivedi S. L. Ceccarelli S. Blair M. W. Upadhyaya H. D. Are A. K. Ortiz R. (2016). Landrace germplasm for improving yield and abiotic stress adaptation. *Trends Plant Sci.* 21 31–42. 10.1016/j.tplants.2015.10.012 26559599

[B54] Edwards J. Johnson C. Santos-Medellín C. Lurie E. Podishetty N. K. Bhatnagar S. (2015). Structure, variation, and assembly of the root-associated microbiomes of rice. *Proc. Natl. Acad. Sci. U.S.A.* 112 E911–E920. 10.1073/pnas.1414592112 25605935 PMC4345613

[B55] Ellouze W. Hamel C. Singh A. K. Mishra V. DePauw R. M. Knox R. E. (2018). Abundance of the arbuscular mycorrhizal fungal taxa associated with the roots and rhizosphere soil of different durum wheat cultivars in the Canadian prairies. *Can. J. Microbiol.* 64 527–536. 10.1139/cjm-2017-0637 29633625

[B56] El-Maraghy S. S. Tohamy T. A. Hussein K. A. (2020). Role of plant-growth promoting fungi (PGPF) in defensive genes expression of *Triticum aestivum* against wilt disease. *Rhizosphere* 15: 100.

[B57] Erginbaş Orakcı G. Morgounov A. Dababat A. A. (2018). Determination of resistance in winter wheat genotypes to the dryland root rots caused by *Fusarium culmorum* in Turkey. *Uluslar Tarım Yaban Hayatı Bilimleri Dergisi* 4 193–202. 10.24180/ijaws.414501

[B58] Fan K. Weisenhorn P. Gilbert J. A. Chu H. (2018). Wheat rhizosphere harbors a less complex and more stable microbial co-occurrence pattern than bulk soil. *Soil Biol. Biochem.* 125 251–260. 10.1016/j.soilbio.2018.07.022

[B59] Feldman M. Kislev M. E. (2007). Domestication of emmer wheat and evolution of free-threshing tetraploid wheat. *Isr. J. Plant Sci.* 55 207–221.

[B60] Finkel O. M. Castrillo G. Herrera Paredes S. Salas González I. Dangl J. L. (2017). Understanding and exploiting plant beneficial microbes. *Curr. Opin. Plant Biol.* 38 155–163. 10.1016/j.pbi.2017.04.018 28622659 PMC5561662

[B61] Finlay B. J. Maberly S. C. Cooper J. I. (1997). Microbial diversity and ecosystem function. *Oikos* 80 209–213.

[B62] Fitzpatrick C. R. Copeland J. Wang P. W. Guttman D. S. Kotanen P. M. Johnson M. T. J. (2018). Assembly and ecological function of the root microbiome across angiosperm plant species. *Proc. Natl. Acad. Sci. U.S.A.* 115 E1157–E1165. 10.1073/pnas.1717617115 29358405 PMC5819437

[B63] Fox A. R. Soto G. Valverde C. Russo D. Lagares A. Zorreguieta Á (2016). Major cereal crops benefit from biological nitrogen fixation when inoculated with the nitrogen-fixing bacterium *Pseudomonas protegens* Pf-5 X. *Environ. Microbiol* 18 3522–3534. 10.1111/1462-2920.13376 27198923

[B64] Fricano A. Brandolini A. Rossini L. Sourdille P. Wunder J. Effgen S. (2014). Crossability of *Triticum urartu* and *Triticum monococcum* wheats, homoeologous recombination, and description of a panel of interspecific introgression lines. *G3* 4 1931–1941. 10.1534/g3.114.013623 25147190 PMC4199699

[B65] Fuhrman J. A. (2009). Microbial community structure and its functional implications. *Nature* 459 193–199. 10.1038/nature08058 19444205

[B66] Gajda A. M. Czyż E. A. Stanek-Tarkowska J. Furtak K. M. Grządziel J. (2017). Effects of long-term tillage practices on the quality of soil under winter wheat. *Plant Soil Environ.* 63 236–242. 10.17221/223/2017-pse

[B67] Gantar M. Kerby N. W. Rowell P. (1993). Colonization of wheat (*Triticum vulgare* L.) by N_2_-fixing cyanobacteria: III. The role of a hormogonia-promoting factor. *New Phytol.* 124 505–513. 10.1111/j.1469-8137.1993.tb03842.x 33874558

[B68] Ganugi P. Masoni A. Pietramellara G. Benedettelli S. (2019). A review of studies from the last twenty years on plant–arbuscular mycorrhizal associations and their uses for wheat crops. *Agronomy* 9: 112.

[B69] Gayon J. Zallen D. T. (1998). The role of the Vilmorin Company in the promotion and diffusion of the experimental science of heredity in France, 1840-1J. *Hist. Biol.* 31 241–262. 10.1023/a:1004335619901 11620305

[B70] George T. S. French A. S. Brown L. K. Karley A. J. White P. J. Ramsay L. (2014). Genotypic variation in the ability of landraces and commercial cereal varieties to avoid manganese deficiency in soils with limited manganese availability: is there a role for root-exuded phytases? *Physiol. Plant.* 151 243–256. 10.1111/ppl.12151 24438182

[B71] Germida J. J. Siciliano S. D. Renato de Freitas J. Seib A. M. (1998). Diversity of root-associated bacteria associated with field-grown canola (*Brassica napus* L.) and wheat (*Triticum aestivum* L.). *FEMS Microbiol. Ecol.* 26 43–50. 10.1111/j.1574-6941.1998.tb01560.x

[B72] Germida J. Siciliano S. (2001). Taxonomic diversity of bacteria associated with the roots of modern, recent and ancient wheat cultivars. *Biol. Fertil. Soil.* 33 410–415. 10.1007/s003740100343

[B73] Gill B. S. Appels R. Botha-Oberholster A. M. Buell C. R. Bennetzen J. L. Chalhoub B. (2004). A workshop report on wheat genome sequencing: international genome research on wheat consortium. *Genetics* 168 1087–1096. 10.1534/genetics.104.034769 15514080 PMC1448818

[B74] Gill S. Abid M. Azam F. (2006). Root-induced changes in potential nitrification and nitrate reductase activity of the rhizospheric soil of wheat (*Triticum aestivum* l.) and chickpea (*Cicer arietinum* l.). *Pak. J. Bot.* 38:991.

[B75] Glémin S. Scornavacca C. Dainat J. Burgarella C. Viader V. Ardisson M. (2019). Pervasive hybridizations in the history of wheat relatives. *Sci. Adv.* 5:eaav9188. 10.1126/sciadv.aav9188 31049399 PMC6494498

[B76] Glick B. R. (2005). Modulation of plant ethylene levels by the bacterial enzyme ACC deaminase. *FEMS Microbiol. Lett.* 251 1–7. 10.1016/j.femsle.2005.07.030 16099604

[B77] Glick B. R. (2014). Bacteria with ACC deaminase can promote plant growth and help to feed the world. *Microbiol Res.* 169 30–39. 10.1016/j.micres.2013.09.009 24095256

[B78] Glover N. M. (2016). Homoeologs: what are they and how do we infer them? *Trends Plant Sci.* 21 609–621. 10.1016/j.tplants.2016.02.005 27021699 PMC4920642

[B79] Golan G. Hendel E. Espitia G. E. M. Schwartz N. Peleg Z. (2018). Activation of seminal root primordia during wheat domestication reveals underlying mechanisms of plant resilience. *Plant Cell Environ.* 41 755–766. 10.1111/pce.13138 29320605

[B80] Golizadeh V. M. Dashti H. Riseh R. S. Bihamta M. R. (2017). Screening bread wheat germplasm for resistance to take-all disease (*Gaeumannomyces graminis* var. tritici) in greenhouse conditions. *J. Agric. Sci. Tech.* 19 1173–1184.

[B81] Gqozo M. P. Bill M. Siyoum N. Labuschagne N. Korsten L. (2020). Fungal diversity and community composition of wheat rhizosphere and non-rhizosphere soils from three different agricultural production regions of South Africa. *Appl. Soil Ecol.* 151: 103543. 10.1016/j.apsoil.2020.103543

[B82] Gu Y. H. Mazzola M. (2003). Modification of fluorescent pseudomonad community and control of apple replant disease induced in a wheat cultivar-specific manner. *Appl. Soil Ecol.* 24 57–72. 10.1016/s0929-1393(03)00066-0

[B83] Guo X. Zhou X. Hale L. Yuan M. Feng J. Ning D. (2018). Taxonomic and functional responses of soil microbial communities to annual removal of aboveground plant biomass. *Front. Microbiol.* 9:954. 10.3389/fmicb.2018.00954 29904372 PMC5990867

[B84] Gyaneshwar P. Naresh Kumar G. Parekh L. J. Poole P. S. (2002). Role of soil microorganisms in improving P nutrition of plants. *Plant Soil* 245 83–93.

[B85] Haas M. Schreiber M. Mascher M. (2019). Domestication and crop evolution of wheat and barley: genes, genomics, and future directions. *J. Integr. Plant Biol.* 61 204–225. 10.1111/jipb.12737 30414305

[B86] Haberer G. Mayer K. F. Spannagl M. (2016). The big five of the monocot genomes. *Curr. Opin. Plant Biol.* 30 33–40. 10.1016/j.pbi.2016.01.004 26866569

[B87] Hagn A. Pritsch K. Schloter M. Munch J. C. (2003). Fungal diversity in agricultural soil under different farming management systems, with special reference to biocontrol strains of *Trichoderma* spp. *Biol. Fertil. Soil.* 38 236–244.

[B88] Haichar F. Z. Heulin T. Guyonnet J. P. Achouak W. (2016). Stable isotope probing of carbon flow in the plant holobiont. *Curr. Opin. Biotechnol* 41 9–13. 10.1016/j.copbio.2016.02.023 27019410

[B89] Haichar F. Z. Marol C. Berge O. Rangel-Castro J. I Prosser J. I Balesdent J. (2008). Plant host habitat and root exudates shape soil bacterial community structure. *ISME J* 2 1221–1230. 10.1038/ismej.2008.80 18754043

[B90] Hamel P. Saint-Georges Y. Pinto B. D. Lachacinski N. Altamura N. Dujardin G. (2004). Redundancy in the function of mitochondrial phosphate transport in *Saccharomyces cerevisiae* and *Arabidopsis thaliana*. *Mol. Microbiol* 51 307–317. 10.1046/j.1365-2958.2003.03810.x 14756774

[B91] Hamonts K. Clough T. J. Stewart A. Clinton P. W. Richardson A. E. Wakelin S. A. (2013). Effect of nitrogen and waterlogging on denitrifier gene abundance, community structure and activity in the rhizosphere of wheat. *FEMS Microbiol. Ecol.* 83 568–584. 10.1111/1574-6941.12015 23006139

[B92] Hassan W. Bano R. Bashir F. David J. (2014). Comparative effectiveness of ACC-deaminase and/or nitrogen-fixing rhizobacteria in promotion of maize (*Zea mays* L.) growth under lead pollution. *Environ. Sci. Pollut. Res.* 21 10983–10996. 10.1007/s11356-014-3083-5 24888619

[B93] Hassani M. A. Duran P. Hacquard S. (2018). Microbial interactions within the plant holobiont. *Microbiome* 6:58. 10.1186/s40168-018-0445-0 29587885 PMC5870681

[B94] Hassani M. A. Özkurt E. Franzenburg S. Stukenbrock E. (2020). Ecological assembly processes of the bacterial and fungal microbiota of wild and domesticated wheat species. *Phytobiomes J.* 4 217–224. 10.1094/pbiomes-01-20-0001-sc

[B95] Hayashi K. Tokida T. Kajiura M. Yanai Y. Yano M. (2015). Cropland soil–plant systems control production and consumption of methane and nitrous oxide and their emissions to the atmosphere. *Soil Sci. Plant Nutr.* 61 2–33.

[B96] Herridge D. F. Peoples M. B. Boddey R. M. (2008). Global inputs of biological nitrogen fixation in agricultural systems. *Plant Soil* 311 1–18. 10.1007/s11104-008-9668-3

[B97] Hetrick B. A. D. Wilson G. W. T. Cox T. S. (1992). Mycorrhizal dependence of modern wheat varieties, landraces, and ancestors. *Can. J. Bot.* 70 2032–2518. 10.1139/b92-253

[B98] Higginbotham R. W. Paulitz T. C. Campbell K. G. Kidwell K. K. (2004). Evaluation of adapted wheat cultivars for tolerance to Pythium root rot. *Plant Dis.* 88 1027–1032. 10.1094/PDIS.2004.88.9.1027 30812217

[B99] Hiltner L. (1904). Über neuere Erfahrungen und Probleme auf dem Gebiete der Bodenbakteriologie unter besonderer Berücksichtigung und Brache. *Arb DLG* 98 59–78.

[B100] Hilton S. Bennett A. J. Chandler D. Mills P. Bending G. D. (2018). Preceding crop and seasonal effects influence fungal, bacterial and nematode diversity in wheat and oilseed rape rhizosphere and soil. *Appl. Soil Ecol.* 126 34–46. 10.1016/j.apsoil.2018.02.007

[B101] Hodgson S. de Cates C. Hodgson J. Morley N. J. Sutton B. C. Gange A. C. (2014). Vertical transmission of fungal endophytes is widespread in forbs. *Ecol. Evol.* 4 1199–1208. 10.1002/ece3.953 24834319 PMC4020682

[B102] Houlden A. Timms-Wilson T. M. Day M. J. Bailey M. J. (2008). Influence of plant developmental stage on microbial community structure and activity in the rhizosphere of three field crops. *FEMS Microbiol. Ecol.* 65 193–201. 10.1111/j.1574-6941.2008.00535.x 18616582

[B103] Huang M. Sanchez-Moreiras A. M. Abel C. Sohrabi R. Lee S. Gershenzon J. (2012). The major volatile organic compound emitted from *Arabidopsis thaliana* flowers, the sesquiterpene (E)−β−caryophyllene, is a defense against a bacterial pathogen. *New Phytol.* 193 997–1008. 10.1111/j.1469-8137.2011.04001.x 22187939

[B104] Iannucci A. Canfora L. Nigro F. De Vita P. Beleggia R. (2021). Relationships between root morphology, root exudate compounds and rhizosphere microbial community in durum wheat. *Appl. Soil Ecol.* 158 103781. 10.1016/j.apsoil.2020.103781

[B105] Imperiali N. Chiriboga X. Schlaeppi K. Fesselet M. Villacrés D. Jaffuel G. (2017). Combined field inoculations of *Pseudomonas* bacteria, arbuscular mycorrhizal fungi, and entomopathogenic nematodes and their effects on wheat performance. *Front. Plant Sci.* 8:1809. 10.3389/fpls.2017.01809 29163562 PMC5671467

[B106] Ingram D. M. Cook R. J. (1990). Pathogenicity of four *Pythium* species to wheat, barley, peas and lentils. *Plant Pathol.* 39 110–117. 10.1111/j.1365-3059.1990.tb02481.x

[B107] International Wheat Genome Sequencing Consortium [IWGSC], Appels R. Eversole K. Stein N. Feuillet C. Keller B. (2018). Shifting the limits in wheat research and breeding using a fully annotated reference genome. *Science* 361:eaar7191. 10.1126/science.aar7191 30115783

[B108] Jacoby R. Peukert M. Succurro A. Koprivova A. Kopriva S. (2017). The role of soil microorganisms in plant mineral nutrition—Current knowledge and future directions. *Front. Plant Sci.* 8:1617. 10.3389/fpls.2017.01617 28974956 PMC5610682

[B109] Janzen H. H. (1990). Deposition of nitrogen into the rhizosphere by wheat roots. *Soil Biol. Biochem.* 22 1155–1160. 10.1111/nph.13966 27101777

[B110] Jia J. Zhao S. Kong X. Li Y. Zhao G. He W. (2013). *Aegilops tauschii* draft genome sequence reveals a gene repertoire for wheat adaptation. *Nature* 496 91–95. 10.1038/nature12028 23535592

[B111] Jog R. Pandya M. Nareshkumar G. Rajkumar S. (2014). Mechanism of phosphate solubilization and antifungal activity of *Streptomyces* spp. isolated from wheat roots and rhizosphere and their application in improving plant growth. *Microbiology* 160 778–788. 10.1099/mic.0.074146-0 24430493

[B112] Juhnke M. E. Mathre D. E. Sands D. C. (1987). Identification and characterization of rhizosphere-competent bacteria of wheat. *Appl. Environ. Microbiol.* 53 2793–2799. 10.1128/aem.53.12.2793-2799.1987 16347496 PMC204200

[B113] Kang X. Zhang W. Cai X. Zhu T. Xue Y. Liu C. (2018). *Bacillus velezensis* CC09: a potential ‘vaccine’ for controlling wheat diseases. *Mol. Plant-Microbe Interact.* 31 623–632. 10.1094/MPMI-09-17-0227-R 29372814

[B114] Kapulnik Y. Kushnir U. (1991). Growth dependency of wild, primitive and modern cultivated wheat lines on vesicular-arbuscular mycorrhiza fungi. *Euphytica* 56 27–36. 10.1007/bf00041740

[B115] Kavamura V. N. Hayat R. Clark I. M. Rossmann M. Mendes R. Hirsch P. R. (2018). Inorganic nitrogen application affects both taxonomical and predicted functional structure of wheat rhizosphere bacterial communities. *Front. Microbiol.* 9:1074. 10.3389/fmicb.2018.01074 29896167 PMC5986887

[B116] Kavamura V. N. Mendes R. Bargaz A. Mauchline T. H. (2021). Defining the wheat microbiome: Towards microbiome-facilitated crop production. *Comput. Struct. Biotechnol. J.* 19 1200–1213. 10.1016/j.csbj.2021.01.045 33680361 PMC7902804

[B117] Kavamura V. N. Robinson R. J. Hughes D. Clark I. Rossmann M. Melo I. S. (2020). Wheat dwarfing influences selection of the rhizosphere microbiome. *Sci. Rep.* 10:1452. 10.1038/s41598-020-58402-y 31996781 PMC6989667

[B118] Kazi N. Deaker R. Wilson N. Muhammad K. Trethowan R. (2016). The response of wheat genotypes to inoculation with *Azospirillum brasilense* in the field. *Field Crop Res.* 196 368–378.

[B119] Keel C. Schnider U. Maurhofer M. Voisard C. Laville J. Burger U. (1992). Suppression of root diseases by *Pseudomonas* fluorescens CHA0: importance of the bacterial secondary metabolite 2,4-diacetylphloroglucinol. *Mol. Plant-Microbe Interact.* 5 4–13. 10.1094/mpmi-5-004

[B120] Kinnunen-Grubb M. Sapkota R. Vignola M. Nunes I. M. Nicolaisen M. (2020). Breeding selection imposed a differential selective pressure on the wheat root-associated microbiome. *FEMS Microbiol. Ecol.* 96:fiaa196. 10.1093/femsec/fiaa196 32970821

[B121] Kiszonas A. M. Morris C. F. (2018). Wheat breeding for quality: a historical review. *Cereal Chem.* 95 17–34.

[B122] Kumar P. Thakur S. Dhingra G. K. Singh A. Pal M. K. Harshvardhan K. (2018). Inoculation of siderophore producing rhizobacteria and their consortium for growth enhancement of wheat plant. *Biocatal. Agric. Biotechnol.* 15 264–269. 10.1016/j.bcab.2018.06.019

[B123] Kundu B. S. Nehra K. Yadav R. Tomar M. (2009). Biodiversity of phosphate solubilizing bacteria in rhizosphere of chickpea, mustard and wheat grown in different regions of Haryana. *Indian J. Microbiol.* 49 120–127. 10.1007/s12088-009-0016-y 23100760 PMC3450143

[B124] Kuzyakov Y. Cheng W. (2001). Photosynthesis controls of rhizosphere respiration and organic matter decomposition. *Soil Biol. Biochem.* 33 1915–1925. 10.1016/s0038-0717(01)00117-1

[B125] Kwak Y. S. Weller D. M. (2013). Take-all of wheat and natural disease suppression: a review. *Plant Pathol. J.* 29 125–135. 10.5423/PPJ.SI.07.2012.0112 25288939 PMC4174779

[B126] Lambers H. Mougel C. Jaillard B. Hinsinger P. (2009). Plant-microbe-soil interactions in the rhizosphere: an evolutionary perspective. *Plant Soil* 321 83–115. 10.3389/fpls.2021.636709 34149744 PMC8211891

[B127] Landa B. B. Mavrodi D. M. Thomashow L. S. Weller D. M. (2003). Interactions between strains of 2,4-diacetylphloroglucinol-producing *Pseudomonas fluorescens* in the rhizosphere of wheat. *Phytopathology* 93 982–994. 10.1094/phyto.2003.93.8.982 18943865

[B128] Latz E. Eisenhauer N. Scheu S. Jousset A. (2015). Plant identity drives the expression of biocontrol factors in a rhizosphere bacterium across a plant diversity gradient. *Funct. Ecol.* 29 1225–1234.

[B129] Lehnert H. Serfling A. Enders M. Friedt W. Ordon F. (2017). Genetics of mycorrhizal symbiosis in winter wheat (*Triticum aestivum*). *New Phytol.* 215 779–791. 10.1111/nph.14595 28517039

[B130] Lemanceau P. Blouin M. Muller D. Moënne-Loccoz Y. (2017). Let the core microbiota be functional. *Trends Plant Sci.* 22 583–595. 10.1016/j.tplants.2017.04.008 28549621

[B131] Li C. Zhou A. Sang T. (2006). Genetic analysis of rice domestication syndrome with the wild annual species *Oryza nivara*. *New Phytol.* 170 185–193. 10.1111/j.1469-8137.2005.01647.x 16539615

[B132] Lindig-Cisneros R. Benrey B. Espinosa-García F. (1997). Phytoalexins, resistance traits, and domestication status in *Phaseolus coccineus* and *Phaseolus lunatus*. *J. Chem. Ecol.* 23 1997–2011.

[B133] Ling H. Q. Zhao S. Liu D. Wang J. Sun H. Zhang C. (2013). Draft genome of the wheat A-genome progenitor *Triticum urartu*. *Nature* 496 87–90. 10.1038/nature11997 23535596

[B134] Liu Y. Zuo S. Xu L. Zou Y. Song W. (2012). Study on diversity of endophytic bacterial communities in seeds of hybrid maize and their parental lines. *Arch. Microbiol.* 194 1001–1012. 10.1007/s00203-012-0836-8 22892578

[B135] Lobell D. B. Cassman K. G. Field C. B. (2009). Crop yield gaps: their importance, magnitudes, and causes. *Annu. Rev. Environ. Resour.* 34 179–204. 10.1111/gcb.13617 28063186

[B136] Louca S. Parfrey L. W. Doebeli M. (2016). Decoupling function and taxonomy in the global ocean microbiome. *Science* 353 1272–1277. 10.1126/science.aaf4507 27634532

[B137] Lu T. Ke M. Peijnenburg W. J. G. M. Zhu Y. Zhang M. Sun L. (2018). Investigation of rhizospheric microbial communities in wheat, barley, and two rice varieties at the seedling stage. *J. Agric. Food Chem.* 66 2645–2653. 10.1021/acs.jafc.7b06155 29474068

[B138] Maccaferri M. Cane’ M. Sanguineti M. C. Salvi S. Colalongo M. C. Massi A. (2014). A consensus framework map of durum wheat (*Triticum durum* desf.) suitable for linkage disequilibrium analysis and genome-wide association mapping. *BMC Genomics* 15:873. 10.1186/1471-2164-15-873 25293821 PMC4287192

[B139] Mahmoudi T. R. Yu J. M. Liu S. Pierson L. S. Pierson E. A. (2019). Drought-stress tolerance in wheat seedlings conferred by phenazine-producing rhizobacteria. *Front. Microbiol.* 10:1590. 10.3389/fmicb.2019.01590 31354678 PMC6636665

[B140] Mahoney A. K. Yin C. Hulbert S. H. (2017). Community structure, species variation, and potential functions of rhizosphere-associated bacteria of different winter wheat (*Triticum aestivum*) cultivars. *Front. Plant Sci.* 8:132. 10.3389/fpls.2017.00132 28243246 PMC5303725

[B141] Majeed A. Abbasi M. K. Hameed S. Imran A. Rahim N. (2015). Isolation and characterization of plant growth-promoting rhizobacteria from wheat rhizosphere and their effect on plant growth promotion. *Front. Microbiol.* 6:198. 10.3389/fmicb.2015.00198 25852661 PMC4362341

[B142] Maketon C. Fortuna A.-M. Okubara P. A. (2012). Cultivar-dependent transcript accumulation in wheat roots colonized by *Pseudomonas* fluorescens Q8r1-96 wild type and mutant strains. *Biol. Control* 60, 216–224. 10.1016/j.biocontrol.2011.11.002

[B143] Mao Y. Yannarell A. C. Davis S. C. Mackie R. I. (2013). Impact of different bioenergy crops on N-cycling bacterial and archaeal communities in soil. *Environ. Microbiol.* 15 928–942. 22891790 10.1111/j.1462-2920.2012.02844.x

[B144] Martín-Robles N. Lehmann A. Seco E. Aroca R. Rillig M. C. Milla R. (2018). Impacts of domestication on the arbuscular mycorrhizal symbiosis of 27 crop species. *New Phytol* 218 322–334. 10.1111/nph.14962 29281758

[B145] Matthews A. Pierce S. Hipperson H. Raymond B. (2019). Rhizobacterial community assembly patterns vary between crop species. *Front. Microbiol.* 10:581. 10.3389/fmicb.2019.00581 31019492 PMC6458290

[B146] Mavrodi D. V. Parejko J. A. Mavrodi O. V. Kwak Y. S. Weller D. M. Blankenfeldt W. (2013). Recent insights into the diversity, frequency and ecological roles of phenazines in fluorescent *Pseudomonas* spp. *Environ Microbiol.* 15 675–686. 10.1111/j.1462-2920.2012.02846.x 22882648

[B147] Mavrodi O. V. Walter N. Elateek S. Taylor C. G. Okubara P. A. (2012). Suppression of Rhizoctonia and Pythium root rot of wheat by new strains of *Pseudomonas*. *BioControl* 62 93–102. 10.1016/j.biocontrol.2012.03.013

[B148] Mazzola M. Funnell D. L. Raaijmakers J. M. (2004). Wheat cultivar-specific selection of 2,4-diacetylphloroglucinol-producing fluorescent *Pseudomonas* species from resident soil populations. *Microb. Ecol.* 48 338–348. 10.1007/s00248-003-1067-y 15692854

[B149] Mazzola M. Gu Y.-H. (2002). Wheat genotype-specific induction of soil microbial communities suppressive to disease incited by *Rhizoctonia solani* anastomosis group (AG)-5 and AG-8. *Phytopathology* 92, 1300–1307. 10.1094/PHYTO.2002.92.12.1300 18943884

[B150] McMillan V. E. Gutteridge R. J. Hammond-Kosack K. E. (2014). Identifying variation in resistance to the take-all fungus, *Gaeumannomyces graminis var. tritici*, between different ancestral and modern wheat species. *BMC Plant Biol.* 14:212. 10.1186/s12870-014-0212-8 25084989 PMC4236718

[B151] Meyer J. B. Lutz M. P. Frapolli M. Péchy-Tarr M. Rochat L. Keel C. (2010). Interplay between wheat cultivars, biocontrol pseudomonads, and soil. *Appl. Environ. Microbiol.* 76 6196–6204. 10.1128/AEM.00752-10 20675454 PMC2937482

[B152] Meziani S. Nadaud I. Gaillard-Martinie B. Chambon C. Benali M. Branlard G. (2019). Proteomic analysis of mature kernel aleurone layer of *Triticum spelta* and three wheat related species. *Nutr. Santé* 8 27–35.

[B153] Micallef S. A. Shiaris M. P. Colón-Carmona A. (2009). Influence of accessions on rhizobacterial communities and natural variation in root exudates. *J. Exp. Bot.* 60 1729–1742. 10.1093/jxb/erp053 19342429 PMC2671628

[B154] Mideksa T. Letta T. Bayisa T. Abinasa M. Tilahun A. Hundie B. (2018). Bread wheat varietal development and release in southeastern highlands of Ethiopia. *Am. J. Biol. Environ. Stat.* 4 15–19. 10.11648/j.ajbes.20180401.13

[B155] Moënne-Loccoz Y. Mavingui P. Combes C. Normand P. Steinberg C. (2015). “Microorganisms and biotic interactions,” in *Environmental Microbiology: Fundamentals and Applications*, eds Bertrand J. C. Caumette P. Lebaron P. Matheron R. Normand P. Sime-Ngando T. (Dordrecht: Springer), 395–444. 10.1007/978-94-017-9118-2_11

[B156] Molina-Favero C. Creus C. M. Simontacchi M. Puntarulo S. Lamattina L. (2008). Aerobic nitric oxide production by *Azospirillum brasilense* Sp245 and its influence on root architecture in tomato. *Mol. Plant-Microbe Interact.* 21 1001–1009. 10.1094/MPMI-21-7-1001 18533840

[B157] Mujeeb-Kazi A. (2006). “Utilization of genetic resources for bread wheat improvement,” in *Genetic Resources, Chromosome Engineering, and Crop Improvement*, eds Singh R. J. Jauhar P. P. (Boca Raton, FL: CRC Series), 61–97. 10.1201/9780203489260.ch3

[B158] Mustafa G. Randoux B. Tisserant B. Fontaine J. Magnin-Robert M. Lounès-Hadj Sahraoui A. (2016). Phosphorus supply, arbuscular mycorrhizal fungal species, and plant genotype impact on the protective efficacy of mycorrhizal inoculation against wheat powdery mildew. *Mycorrhiza* 26 685–697. 10.1007/s00572-016-0698-z 27130314

[B159] Narula N. Kumar V. Behl R. K. Deubel A. Gransee A. Merbach W. (2000). Effect of P-solubilizing *Azotobacter chroococcum* on N, P, K uptake in P-responsive wheat genotypes grown under greenhouse conditions. *J. Plant Nutr. Soil Sci.* 163 393–398.

[B160] Naz I. Mirza M. S. Bano A. (2018). Molecular characterization of rhizosphere bacterial communities associated with wheat (*Triticum aestivum* l.) cultivars at flowering stage. *J. Anim. Plant Sci.* 24 1123–1134.

[B161] Newton A. C. Fitt B. D. L. Atkins S. D. Walters D. R. Daniell T. J. (2010). Pathogenesis, parasitism and mutualism in the trophic space of microbe–plant interactions. *Trends Microbiol.* 18 365–373. 10.1016/j.tim.2010.06.002 20598545

[B162] Niu Z. Klindworth D. L. Friesen T. L. Chao S. Jin Y. Cai X. (2011). Targeted introgression of a wheat stem rust resistance gene by DNA marker-assisted chromosome engineering. *Genetics* 187 1011–1021. 10.1534/genetics.110.123588 21242535 PMC3070511

[B163] Nowell R. W. Laue B. E. Sharp P. M. Green S. (2016). Comparative genomics reveals genes significantly associated with woody hosts in the plant pathogen *Pseudomonas syringae*: adaptation to woody hosts in *Pseudomonas syringae*. *Mol. Plant Pathol.* 17 1409–1424. 10.1111/mpp.12423 27145446 PMC5132102

[B164] Nuccio E. E. Starr E. Karaoz U. Brodie E. L. Zhou J. Tringe S. G. (2020). Niche differentiation is spatially and temporally regulated in the rhizosphere. *ISME J.* 14 999–1014. 10.1038/s41396-019-0582-x 31953507 PMC7082339

[B165] O’Sullivan C. A. Fillery I. R. P. Roper M. M. Richards R. A. (2016). Identification of several wheat landraces with biological nitrification inhibition capacity. *Plant Soil* 404 61–74. 10.1007/s11104-016-2822-4

[B166] Ofaim S. Ofek-Lalzar M. Sela N. Jinag J. Kashi Y. Minz D. (2017). Analysis of microbial functions in the rhizosphere using a metabolic-network based framework for metagenomics interpretation. *Front. Microbiol.* 8:1606. 10.3389/fmicb.2017.01606 28878756 PMC5572346

[B167] Ofek-Lalzar M. Gur Y. Ben-Moshe S. Sharon O. Kosman E. Mochli E. (2016). Diversity of fungal endophytes in recent and ancient wheat ancestors *Triticum dicoccoides* and *Aegilops sharonensis*. *FEMS Microbiol. Ecol.* 92 fiw152. 10.1093/femsec/fiw152 27402714

[B168] Okubara P. A. Bonsall R. F. (2008). Accumulation of *Pseudomonas*-derived 2,4-diacetylphloroglucinol on wheat seedling roots is influenced by host cultivar. *Biol. Control* 46, 322–331. 10.1016/j.biocontrol.2008.03.013

[B169] Okubara P. A. Call D. R. Kwak Y. Skinner D. Z. (2010). Induction of defense gene homologues in wheat roots during interactions with *Pseudomonas fluorescens*. *BioControl* 55 118–125.

[B170] Oros G. Naár Z. Magyar D. (2013). Susceptibility of wheat varieties to soil-borne *Rhizoctonia* infection. *Am. J. Plant Sci.* 4:2.

[B171] Osborne S. J. McMillan V. E. White R. Hammond-Kosack K. E. (2018). Elite UK winter wheat cultivars differ in their ability to support the colonization of beneficial root-infecting fungi. *J. Exp. Bot.* 69 3103–3115. 10.1093/jxb/ery136 29648609 PMC5972604

[B172] Özkan H. Willcox G. Graner A. Salamini F. Kilian B. (2011). Geographic distribution and domestication of wild emmer wheat (*Triticum dicoccoides*). *Genet. Resour. Crop Evol.* 58 11–53. 10.1007/s10722-010-9581-5

[B173] Özkurt E. Hassani M. A. Sesiz U. Künzel S. Dagan T. Özkan H. (2020). Seed-derived microbial colonization of wild emmer and domesticated bread wheat (*Triticum dicoccoides* and *Triticum aestivum*) seedlings shows pronounced differences in overall diversity and composition. *mBio* 11:e02637–20. 10.1128/mBio.02637-20 33203759 PMC7683402

[B174] Pagnani G. Galieni A. Stagnari F. Pellegrini M. Del Gallo M. Pisante M. (2020). Open field inoculation with PGPR as a strategy to manage fertilization of ancient *Triticum* genotypes. *Biol. Fertil. Soils* 56, 111–124. 10.1007/s00374-019-01407-1

[B175] Parnell J. J. Berka R. Young H. A. Sturino J. M. Kang Y. Barnhart D. M. (2016). From the lab to the farm: an industrial perspective of plant beneficial microorganisms. *Front. Plant Sci.* 7:1. 10.3389/fpls.2016.01110 27540383 PMC4973397

[B176] Peleg Z. Fahima T. Korol A. B. Abbo S. Saranga Y. (2011). Genetic analysis of wheat domestication and evolution under domestication. *J. Exp. Bot.* 62 5051–5061. 10.1093/jxb/err206 21778183 PMC3193012

[B177] Pellegrino E. Öpik M. Bonari E. Ercoli L. (2015). Responses of wheat to arbuscular mycorrhizal fungi: a meta-analysis of field studies from 1975 to 2013. *Soil Biol. Biochem.* 84 210–217.

[B178] Peng J. Ronin Y. Fahima T. Roder M. S. Li Y. Nevo E. (2003). Domestication quantitative trait loci in *Triticum dicoccoides*, the progenitor of wheat. *Proc. Natl. Acad. Sci. U.S.A.* 100 2489–2494. 10.1073/pnas.252763199 12604784 PMC151368

[B179] Pérez-de-Luque A. Tille S. Johnson I. Pascual-Pardo D. Ton J. Cameron D. D. (2017). The interactive effects of arbuscular mycorrhiza and plant growth-promoting rhizobacteria synergistically enhance host plant defences against pathogens. *Sci. Rep.* 7:16409. 10.1038/s41598-017-16697-4 29180695 PMC5703727

[B180] Pérez-Jaramillo J. E. Carrión V. J. de Hollander M. Raaijmakers J. M. (2018). The wild side of plant microbiomes. *Microbiome* 6:143. 10.1186/s40168-018-0519-z 30115122 PMC6097318

[B181] Perin L. Martínez-Aguilar L. Castro-González R. Santos P. E. Cabellos-Avelar T. Guedes H. V. (2006). Diazotrophic *Burkholderia* species associated with field-grown maize and sugarcane. *Appl. Environ. Microbiol.* 72 3103–3110. 10.1128/AEM.72.5.3103-3110.2006 16672447 PMC1472400

[B182] Pestsova E. G. Börner A. Röder M. S. (2005). Development and QTL assessment of *Triticum aestivum*–*Aegilops tauschii* introgression lines. *Theor. Appl. Genet.* 112:634. 10.1007/s00122-005-0166-1 16341683

[B183] Philippot L. Raaijmakers J. M. Lemanceau P. van der Putten W. H. (2013). Going back to the roots: the microbial ecology of the rhizosphere. *Nat. Rev. Microbiol.* 11 789–799. 10.1038/nrmicro3109 24056930

[B184] Pont C. Leroy T. Seidel M. Tondelli A. Duchemin W. Armisen D. (2019). Tracing the ancestry of modern bread wheats. *Nat. Genet.* 51 905–911. 10.1038/s41588-019-0393-z 31043760

[B185] Prudence S. M. M. Newitt J. T. Worsley S. F. Macey M. C. Murrell J. C. Lehtovirta-Morley L. E. (2021). Soil, senescence and exudate utilisation: characterisation of the Paragon var. spring bread wheat root microbiome. *Environ. Microbiome* 16:12. 10.1186/s40793-021-00381-2 34154664 PMC8215762

[B186] Raaijmakers J. M. Paulitz T. C. Steinberg C. Alabouvette C. Moënne-Loccoz Y. (2009). The rhizosphere: a playground and battlefield for soilborne pathogens and beneficial microorganisms. *Plant Soil* 321 341–361. 10.1007/s11104-008-9568-6

[B187] Rascovan N. Carbonetto B. Perrig D. Díaz M. Canciani W. Abalo M. (2016). Integrated analysis of root microbiomes of soybean and wheat from agricultural fields. *Sci. Rep.* 6 1–12. 10.1038/srep28084 27312589 PMC4911569

[B188] Reinhold-Hurek B. Hurek T. (2011). Living inside plants: bacterial endophytes. *Curr. Opin. Plant Biol.* 14 435–443. 10.1016/j.pbi.2011.04.004 21536480

[B189] Reinhold-Hurek B. Bünger W. Burbano C. S. Sabale M. Hurek T. (2015). Roots shaping their microbiome: global hotspots for microbial activity. *Annu. Rev. Phytopathol.* 53 403–424. 10.1146/annurev-phyto-082712-102342 26243728

[B190] Rilling J. I. Acuña J. J. Sadowsky M. J. Jorquera M. A. (2018). Putative nitrogen-fixing bacteria associated with the rhizosphere and root endosphere of wheat plants grown in an andisol from southern Chile. *Front. Microbiol.* 9:2710. 10.3389/fmicb.2018.02710 30524385 PMC6256256

[B191] Rong J. K. Millet E. Manisterski J. Feldman M. (2000). A new powdery mildew resistance gene: Introgression from wild emmer into common wheat and RFLP-based mapping. *Euphytica* 115 121–126.

[B192] Rossmann M. Chiaramonte B. Dumack K. Fiore-Donno A. M. Mendes L. W. Raaijmakers J. M. (2020). Multitrophic interactions in the rhizosphere microbiome of wheat: from bacteria and fungi to protists. *FEMS Microbiol. Ecol.* 96:fiaa032. 10.1093/femsec/fiaa032 32124916

[B193] Roucou A. Violle C. Fort F. Roumet P. Ecarnot M. Vile D. (2018). Shifts in plant functional strategies over the course of wheat domestication. *J. Appl. Ecol.* 55 25–37. 10.1111/1365-2664.13029

[B194] Saia S. Fragasso M. De Vita P. Beleggia R. (2019). Metabolomics provides valuable insight for the study of durum wheat: a review. *J. Agric. Food Chem.* 67 3069–3085. 10.1021/acs.jafc.8b07097 30829031

[B195] Salamini F. Özkan H. Brandolini A. Schäfer-Pregl R. Martin W. (2002). Genetics and geography of wild cereal domestication in the near east. *Nat. Rev. Genet.* 3 429–441. 10.1038/nrg817 12042770

[B196] Salem G. Stromberger M. E. Byrne P. F. Manter D. K. El-Feki W. Weir T. L. (2018). Genotype-specific response of winter wheat (*Triticum aestivum* L.) to irrigation and inoculation with ACC deaminase bacteria. *Rhizosphere* 8 1–7. 10.1016/j.rhisph.2018.08.001

[B197] Santi C. Bogusz D. Franche C. (2013). Biological nitrogen fixation in non-legume plants. *Ann. Bot.* 111 743–767. 10.1093/aob/mct048 23478942 PMC3631332

[B198] Sato K. Jiang H. Y. (1996a). Gram-positive bacterial flora on the root surface of wheat (*Triticum aestivum* L.) grown under different soil conditions. *Biol. Fertil. Soils* 23 121–125. 10.1007/s003740050148

[B199] Sato K. Jiang H. Y. (1996b). Gram-negative bacterial flora on the root surface of wheat (*Triticum aestivum*) grown under different soil conditions. *Biol. Fertil. Soils* 23 273–281. 10.1007/bf00335955

[B200] Schloter M. Hartmann A. (1998). Endophytic and surface colonization of wheat roots (*Triticum aestivum*) by different *Azospirillum brasilense* strains studied with strain-specific monoclonal antibodies. *Symbiosis* 25 159–179.

[B201] Schulz B. Boyle C. (2006). “What are endophytes?,” in *Microbial Root Endophytes*, eds Schulz B. J. E. Boyle C. J. C. Sieber T. N. (Berlin: Springer), 1–13. 10.1007/3-540-33526-9_1

[B202] Seguel A. Castillo C. G. Morales A. Campos P. Cornejo P. Borie F. (2016). Arbuscular mycorrhizal symbiosis in four Al-tolerant wheat genotypes grown in an acidic Andisol. *J. Soil Sci. Plant Nutr.* 16 164–173.

[B203] Shakir M. A. Bano A. Arshad M. (2012). Rhizosphere bacteria containing ACC-deaminase conferred drought tolerance in wheat grown under semi-arid climate. *Soil Environ.* 31 108–112.

[B204] Shaposhnikov A. I. Morgounov A. I. Akin B. Makarova N. M. Belimov A. A. Tikhonovich I. A. (2016). Comparative characteristics of root systems and root exudation of synthetic, landrace and modern wheat varieties. *Agric. Biol.* 51 68–78.

[B205] Sheng X. F. He L. Y. (2011). Solubilization of potassium-bearing minerals by a wild-type strain of *Bacillus edaphicus* and its mutants and increased potassium uptake by wheat. *Can. J. Microbiol.* 52 56–72. 10.1139/w05-117 16541160

[B206] Shi S. Chang J. Tian L. Nasir F. Ji L. Li X. (2019). Comparative analysis of the rhizomicrobiome of the wild versus cultivated crop: insights from rice and soybean. *Arch. Microbiol.* 201 879–888. 10.1007/s00203-019-01638-8 30963196

[B207] Siciliano S. D. Theoret C. M. de Freitas J. R. Hucl P. J. Germida J. J. (1998). Differences in the microbial communities associated with the roots of different cultivars of canola and wheat. *Can. J. Microbiol.* 44 844–851. 10.1139/w98-075

[B208] Simon J. C. Marchesi J. R. Mougel C. Selosse M.-A. (2019). Host-microbiota interactions: from holobiont theory to analysis. *Microbiome* 7: 5. 10.1186/s40168-019-0619-4 30635058 PMC6330386

[B209] Simonin M. Dasilva C. Terzi V. Ngonkeu E. L. M. Diouf D. Kane A. (2020). Influence of plant genotype and soil on the wheat rhizosphere microbiome: evidences for a core microbiome across eight African and European soils. *FEMS Microbiol. Ecol.* 96:fiaa067. 10.1093/femsec/fiaa067 32275297

[B210] Smit E. Leeflang P. Glandorf B. van Elsas J. D. Wernars K. (1999). Analysis of fungal diversity in the wheat rhizosphere by sequencing of cloned PCR-amplified genes encoding 18S rRNA and temperature gradient gel electrophoresis. *Appl. Environ. Microbiol.* 65 2614–2621. 10.1128/AEM.65.6.2614-2621.1999 10347051 PMC91386

[B211] Somenahally A. DuPont J. I. Brady J. McLawrence J. Northup B. Gowda P. (2018). Microbial communities in soil profile are more responsive to legacy effects of wheat-cover crop rotations than tillage systems. *Soil Biol. Biochem.* 123 126–135. 10.1016/j.soilbio.2018.04.025

[B212] Soriano J. M. Villegas D. Aranzana M. J. del Moral L. F. G. Royo C. (2016). Genetic structure of modern durum wheat cultivars and mediterranean landraces matches with their agronomic performance. *PLoS One* 11:e0160983. 10.1371/journal.pone.0160983 27513751 PMC4981446

[B213] Spaepen S. Vanderleyden J. Remans R. (2007). Indole-3-acetic acid in microbial and microorganism-plant signaling. *FEMS Microbiol. Rev.* 31 425–448. 10.1111/j.1574-6976.2007.00072.x 17509086

[B214] Spor A. Roucou A. Mounier A. Bru D. Breuil M.-C. Fort F. (2020). Domestication-driven changes in plant traits associated with changes in the assembly of the rhizosphere microbiota in tetraploid wheat. *Sci. Rep.* 10:12234. 10.1038/s41598-020-69175-9 32699344 PMC7376052

[B215] Šramková Z. Gregová E. Šturdík E. (2009). Chemical composition and nutritional quality of wheat grain. *Acta Chimi. Slov.* 2 115–138.

[B216] Stromberger M. E. Abduelafez I. Byrne P. Canela M. M. Elamari A. A. Manter D. K. (2017). Genotype-specific enrichment of 1-aminocyclopropane-1-carboxylic acid deaminase-positive bacteria in winter wheat rhizospheres. *Soil Sci. Soc. Am. J.* 81 796–805. 10.2136/sssaj2016.12.0437

[B217] Szoboszlay M. Näther A. Liu B. Carrillo A. Castellanos T. Smalla K. (2019). Contrasting microbial community responses to salinization and straw amendment in a semiarid bare soil and its wheat rhizosphere. *Sci. Rep.* 9:9795. 10.1038/s41598-019-46070-6 31278291 PMC6611862

[B218] Tian Z. Wang J. W. Li J. Han B. (2021). Designing future crops: challenges and strategies for sustainable agriculture. *Plant J.* 105 1165–1178. 10.1111/tpj.15107 33258137

[B219] Tidiane Sall A. Chiari T. Legesse W. Seid-Ahmed K. Ortiz R. van Ginkel M. (2019). Durum wheat (*Triticum durum* desf.): origin, cultivation and potential expansion in Sub-Saharan Africa. *Agronomy* 9:263.

[B220] Tkacz A. Pini F. Turner T. R. Bestion E. Simmonds J. Howell P. (2020). Agricultural selection of wheat has been shaped by plant-microbe interactions. *Front. Microbiol.* 11:132. 10.3389/fmicb.2020.00132 32117153 PMC7015950

[B221] Torsvik V. Øvreås L. (2002). Microbial diversity and function in soil: from genes to ecosystems. *Curr. Opin. Microbiol.* 5 240–245. 10.1016/s1369-5274(02)00324-7 12057676

[B222] Trebbi D. Maccaferri M. de Heer P. Sørensen A. Giuliani S. Salvi S. (2011). High-throughput SNP discovery and genotyping in durum wheat (*Triticum durum* desf.). *Theor. Appl. Genet.* 123 555–569. 10.1007/s00122-011-1607-7 21611761

[B223] Truyens S. Weyens N. Cuypers A. Vangronsveld J. (2015). Bacterial seed endophytes: genera, vertical transmission and interaction with plants: bacterial seed endophytes. *Environ. Microbiol. Rep.* 7 40–50. 10.3389/fmicb.2019.02659 31798570 PMC6865467

[B224] Turner T. R. Ramakrishnan K. Walshaw J. Heavens D. Alston M. Swarbreck D. (2013). Comparative metatranscriptomics reveals kingdom level changes in the rhizosphere microbiome of plants. *ISME J.* 7 2248–2258. 10.1038/ismej.2013.119 23864127 PMC3834852

[B225] Vacheron J. Desbrosses G. Bouffaud M. L. Touraine B. Moënne-Loccoz Y. Muller D. (2013). Plant growth-promoting rhizobacteria and root system functioning. *Front. Plant Sci.* 4:356. 10.3389/fpls.2013.00356 24062756 PMC3775148

[B226] Valente J. Gerin F. Le Gouis J. Moënne-Loccoz Y. Prigent–Combaret C. (2020). Ancient wheat varieties have a higher ability to interact with plant growth-promoting rhizobacteria. *Plant Cell Environ* 43 246–260. 10.1111/pce.13652 31509886

[B227] Van Dommelen A. Croonenborghs A. Spaepen S. Vanderleyden J. (2009). Wheat growth promotion through inoculation with an ammonium-excreting mutant of *Azospirillum brasilense*. *Biol. Fertil. Soils* 45 549–553. 10.1007/s00374-009-0357-z

[B228] Vandenkoornhuyse P. Quaiser A. Duhamel M. Van A. L. Dufresne A. (2015). The importance of the microbiome of the plant holobiont. *New Phytol.* 206 1196–1206. 10.1111/nph.13312 25655016

[B229] Venieraki A. Dimou M. Pergalis P. Kefalogianni I. Chatzipavlidis I. Katinakis P. (2011). The genetic diversity of culturable nitrogen-fixing bacteria in the rhizosphere of wheat. *Microb. Ecol.* 61 277–285. 10.1007/s00248-010-9747-x 20857096

[B230] Waines J. G. Ehdaie B. (2007). Domestication and crop physiology: roots of green-revolution wheat. *Ann. Bot.* 100 991–998. 10.1093/aob/mcm180 17940075 PMC2759207

[B231] Wang C. Dong D. Wang H. Müller K. Qin Y. Wang H. (2016). Metagenomic analysis of microbial consortia enriched from compost: new insights into the role of *Actinobacteria* in lignocellulose decomposition. *Biotechnol. Biofuel* 9: 22. 10.1186/s13068-016-0440-2 26834834 PMC4731972

[B232] Wang J. Zhang D. Zhang L. Li J. Raza W. Huang Q. (2016). Temporal variation of diazotrophic community abundance and structure in surface and subsoil under four fertilization regimes during a wheat growing season. *Agric. Ecosyst. Environ.* 216 116–124. 10.1016/j.agee.2015.09.039

[B233] Wang Y. Zhao X. Guo Z. Jia Z. Wang S. Ding K. (2018). Response of soil microbes to a reduction in phosphorus fertilizer in rice-wheat rotation paddy soils with varying soil P levels. *Soil Tillage Res.* 181 127–135. 10.1016/j.still.2018.04.005

[B234] Weller D. M. Cook R. J. (1986). “Suppression of root diseases of wheat by fluorescent pseudomonads and mechanisms of action,” in *Iron, Siderophores, and Plant Diseases*, ed. Swinburne T. R. (Boston, MA: Springer), 99–107. 10.1007/978-1-4615-9480-2_12

[B235] Wiese M. V. (1987). *Compendium of Wheat Diseases*, 2nd Edn. St Paul, MN: American Phytopathological Society.

[B236] Wilkinson H. T. Cook R. J. Alldredge J. R. (1985). Relation of inoculum size and concentration to infection of wheat roots by *Gaeumannomyces graminis* var. tritici. *Phytopathology* 75 98–103.

[B237] Wipf H. M. L. Coleman-Derr D. (2021). Evaluating domestication and ploidy effects on the assembly of the wheat bacterial microbiome. *PLoS One* 16:e0248. 10.1371/journal.pone.0248030 33735198 PMC7971525

[B238] Wollersheim R. Trolldenier G. Beringer H. (1987). Effect of bulk density and soil water tension on denitrification in the rhizosphere of spring wheat (*Triticum vulgare*). *Biol. Fertil. Soils* 5 181–187.

[B239] Yang M. Mavrodi D. V. Thomashow L. S. Weller D. M. (2018). Differential response of wheat cultivars to *Pseudomonas brassicacearum* and take-all decline soil. *Phytopathology* 108 1363–1372. 10.1094/PHYTO-01-18-0024-R 29905506 PMC6483097

[B240] York L. M. Carminati A. Mooney S. J. Ritz K. Bennett M. J. (2016). The holistic rhizosphere: integrating zones, processes, and semantics in the soil influenced by roots. *J. Exp. Bot.* 67 3629–3643. 10.1093/jxb/erw108 26980751

[B241] Zahir Z. A. Ghani U. Naveed M. Nadeem S. M. Asghar H. N. (2009). Comparative effectiveness of *Pseudomonas* and *Serratia* sp. containing ACC-deaminase for improving growth and yield of wheat (*Triticum aestivum* L.) under salt-stressed conditions. *Arch. Microbiol.* 191 415–424. 10.1007/s00203-009-0466-y 19255743

[B242] Zhalnina K. Louie K. B. Hao Z. Mansoori N. da Rocha U. N. Shi S. (2018). Dynamic root exudate chemistry and microbial substrate preferences drive patterns in rhizosphere microbial community assembly. *Nat. Microbiol.* 3 470–480. 10.1038/s41564-018-0129-3 29556109

[B243] Zhang L. Du Y. L. Li X. G. (2020). Modern wheat cultivars have greater root nitrogen uptake efficiency than old cultivars. *J. Plant Nutr. Soil Sci.* 183 192–199. 10.1002/jpln.201900353

[B244] Zhu Y. G. Smith S. E. Barritt A. R. Smith F. A. (2001). Phosphorus (P) efficiencies and mycorrhizal responsiveness of old and modern wheat cultivars. *Plant Soil* 237 249–255.

[B245] Zuo S. Li X. Ma Y. Yang S. (2014). Soil microbes are linked to the allelopathic potential of different wheat genotypes. *Plant Soil* 378 49–58.

