# Supplementary material for: Significance of the Diversification of Wheat Species for the Assembly and Functioning of the Root-Associated Microbiome
Source: Front Microbiol. 2022 Jan 4;12:782135. doi: 10.3389/fmicb.2021.782135 (PMC8764353; doi:10.3389/fmicb.2021.782135)
Supplement: Supplementary file 1 [file Presentation_1.pdf]

SUPPLEMENTARY DATA

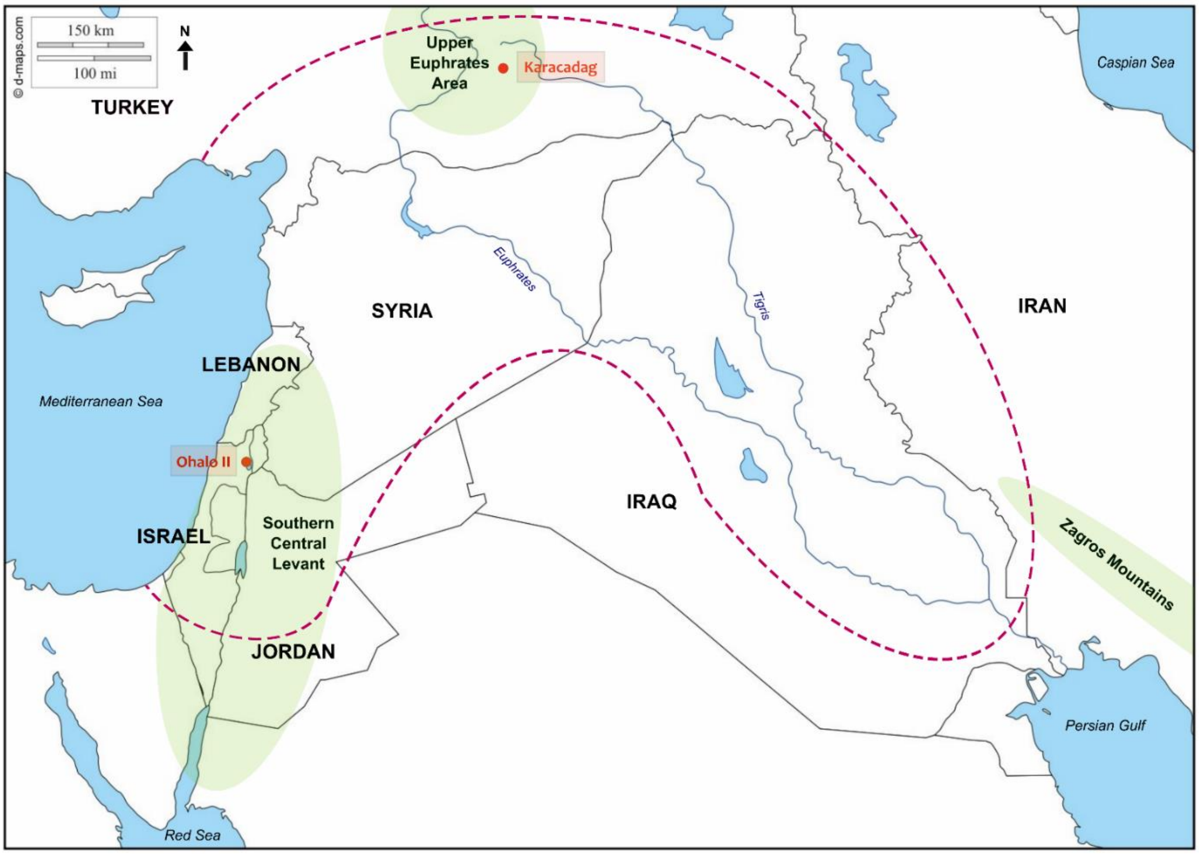

Figure S1. Map of the Fertile Crescent, where wheat domestication took place. The Fertile Crescent is marked by the dashed purple line. Archeobotanical evidence (dated from 10 000 to 12 000 years BP) of cultivated wild cereals were found in the Southern Central Levant, the Upper Euphrates Area and the Zagros Mountains. Karacadag and Ohalo II correspond to key archeological sites where ancient agricultural practices have been evidenced. The northern part of the Fertile Crescent, in Southeast Turkey (which includes the Upper Euphrates Area), is where the earliest domestication events of wild cereals probably took place, 10 500 to 11 000 years BP. Adapted from Salamini *et al.* [27].

Supplementary data 1. List of references cited in Tables 4, S1 and S2

Achouak, W., Abrouk, D., Guyonnet, J., Barakat, M., Ortet, P., Simon, L., Lerondelle, C., Heulin, T., and Haichar, F. Z. (2019). Plant hosts control microbial denitrification activity. *FEMS Microbiol. Ecol.* 95:fiz021.

Almario, J., Moënne-Loccoz, Y., and Muller, D. (2013). Monitoring of the relation between 2,4-diacetylphloroglucinol-producing *Pseudomonas* and *Thielaviopsis basicola* populations by real-time PCR in tobacco black root-rot suppressive and conducive soils. *Soil Biol. Biochem.* 57:144–155.

Bai, L., Cui, J., Jie, W., and Cai, B. (2015). Analysis of the community compositions of rhizosphere fungi in soybeans continuous cropping fields. *Microbiol. Res.* 180:49-56.

Bergkemper, F., Kublik, S., Lang, F., Krüger, J., Vestergaard, G., Schloter, M., and Schulz, S. (2016). Novel oligonucleotide primers reveal a high diversity of microbes which drive phosphorous turnover in soil. *J. Microbiol. Meth.* 125:91–97.

Bokati, D., Herrera, J., and Poudel, R. (2016). Soil Influences colonization of root-associated fungal endophyte communities of maize, wheat, and their progenitors. *J. Mycol.* 2016:1–9.

Bouffaud, M.-L., Renoud, S., Moënne-Loccoz, Y., and Muller, D. (2016). Is plant evolutionary history impacting recruitment of diazotrophs and *nifH* expression in the rhizosphere? *Sci. Rep.* 6:1–9.

Bouffaud, M.-L., Renoud, S., Dubost, A., Moënne-Loccoz, Y., and Muller, D. (2018). 1-Aminocyclopropane-1-carboxylate deaminase producers associated to maize and other *Poaceae* species. *Microbiome* 6:114.

Brisson, V. L., Schmidt, J. E., Northen, T. R., Vogel, J. P., and Gaudin, A. C. M. (2019). Impacts of maize domestication and breeding on rhizosphere microbial community recruitment from a nutrient depleted agricultural soil. *Sci. Rep.* 9:15611.

Bulgarelli, D., Garrido-Oter, R., Münch, P. C., Weiman, A., Dröge, J., Pan, Y., McHardy, A., and Schulze-Lefert, P. (2015). Structure and function of the bacterial root microbiota in wild and domesticated barley. *Cell Host Microbe* 17:392–403.

Bürgmann, H., Widmer, F., Sigler, W. V., and Zeyer, J. (2003). mRNA extraction and reverse transcription-PCR protocol for detection of *nifH* gene expression by *Azotobacter vinelandii* in soil. *Appl. Environ. Microbiol.* 69:1928–1935.

Chaparro, J. M., Badri, D. V., and Vivanco, J. M. (2014). Rhizosphere microbiome assemblage is affected by plant development. *ISME J.* 8:790–803.

Chen, X., Jiang, N., Chen, Z., Tian, J., Sun, N., Xu, M., and Chen, L. (2017). Response of soil *phoD* phosphatase gene to long-term combined applications of chemical fertilizers and organic materials. *Appl. Soil Ecol.* 119:197–204.

DeCoste, N. J., Gadkar, V. J., and Filion, M. (2011). Relative and absolute quantitative real-time PCR-based quantifications of *hcnC* and *phlD* gene transcripts in natural soil spiked with *Pseudomonas* sp. strain LBUM300. *Appl. Environ. Microbiol.* 77:41–47.

Dennert, F., Imperiali, N., Staub, C., Schneider, J., Laessle, T., Zhang, T., Wittwer, R., van der Heijden, M. Smits, T., Schlaeppi, K., Keel, C., and Maurhofer, M. (2018). Conservation tillage and organic farming induce minor variations in *Pseudomonas* abundance, their antimicrobial function and soil disease resistance. *FEMS Microbiol. Ecol.* 94:fiy075.

Edwards, J., Johnson, C., Santos-Medellín, C., Lurie, E., Podishetty, N. K., Bhatnagar, S., et al. (2015). Structure, variation, and assembly of the root-associated microbiomes of rice. *Proc. Natl. Acad. Sci. USA* 112:911–920.

Fan, K., Weisenhorn, P., Gilbert, J. A., and Chu, H. (2018). Wheat rhizosphere harbors a less complex and more stable microbial co-occurrence pattern than bulk soil. *Soil Biol. Biochem.* 125:251–260.

Gadkar, V. J., and Filion, M. (2013). Development of a versatile TaqMan™ real-time quantitative PCR (RT-qPCR) compliant anchor sequence to quantify bacterial gene transcripts from RNA samples containing carryover genomic DNA. *BMC Biotechnol.* 13:1-11.

Gill, S., Abid, M., and Azam, F. (2006). Root-induced changes in potential nitrification and nitrate reductase activity of the rhizospheric soil of wheat (*Triticum aestivum* l) and chickpea (*Cicer arietinum* l.) *Pak. J Bot.* 38:991.

Gqozo, M. P., Bill, M., Siyoum, N., Labuschagne, N., and Korsten, L. (2020). Fungal diversity and community composition of wheat rhizosphere and non-rhizosphere soils from three different agricultural production regions of South Africa. *Appl. Soil Ecol.*151:103543.

Gu, Y.-H., and Mazzola, M. (2003). Modification of fluorescent pseudomonad community and control of apple replant disease induced in a wheat cultivar-specific manner. *Appl. Soil Ecol.* 24:57-72.

Han, Y., Xu, L., Liu, L., Yi, M., Guo, E., Zhang, A., et al. (2017). Illumina sequencing reveals a rhizosphere bacterial community associated with foxtail millet smut disease suppression. *Plant Soil* 410: 411–421.

Henry, S., Baudoin, E., López-Gutiérrez, J. C., Martin-Laurent, F., Brauman, A., and Philippot, L. (2004). Quantification of denitrifying bacteria in soils by *nirK* gene targeted real-time PCR. *J. Microbiol. Meth.* 59:327–335.

Henry, S., Bru, D., Stres, B., Hallet, S., and Philippot, L. (2006). Quantitative detection of the *nosZ* gene, encoding nitrous oxide reductase, and comparison of the abundances of 16S rRNA, *narG*, *nirK*, and *nosZ* genes in soils. *Appl. Environ. Microbiol.* 72:5181–5189.

Hu, J., Wei, Z., Friman, V.-P., Gu, S., Wang, X., Eisenhauer, N., et al. (2016). Probiotic diversity enhances rhizosphere microbiome function and plant disease suppression. *mBio* 7:e01790-16.

Imperiali, N., Dennert, F., Schneider, J., Laessle, T., Velatta, C., Fesselet, M., et al. (2017). Relationships between root pathogen resistance, abundance and expression of *Pseudomonas* antimicrobial genes, and soil properties in representative swiss agricultural soils. *Front. Plant Sci.* 8:427.

Kazerooni, E. A., Maharachchikumbura, S. S. N., Rethinasamy, V., Al-Mahrouqi, H., and Al-Sadi, A. M. (2017). Fungal diversity in tomato rhizosphere soil under conventional and desert farming systems. *Front. Microbiol.* 8:1462.

Kwak, M.-J., Kong, H. G., Choi, K., Kwon, S.-K., Song, J. Y., Lee, J., et al. (2018). Rhizosphere microbiome structure alters to enable wilt resistance in tomato. *Nat. Biotechnol.* 36:1100–1109.

Lee, S. A., Kim, Y., Kim, J. M., Chu, B., Joa, J.-H., Sang, M. K., et al. (2019). A preliminary examination of bacterial, archaeal, and fungal communities inhabiting different rhizocompartments of tomato plants under real-world environments. *Sci. Rep.* 9:1–15.

Leininger, S., Urich, T., Schloter, M., Schwark, L., Qi, J., Nicol, G. W., et al. (2006). *Archaea* predominate among ammonia-oxidizing prokaryotes in soils. *Nature* 442:806–809.

Li, X. (2017). Microbial activity and community diversity in tobacco rhizospheric soil affected by different pre-crops. *Appl. Ecol. Environ. Res.* 15:15–30.

Li, Y., Wang, L., Yang, L., and Li, H. (2014). Dynamics of rhizosphere properties and antioxidative responses in wheat (*Triticum aestivum* L.) under cadmium stress. *Ecotox. Environ. Safe.* 102:55–61.

Liu, F., Hewezi, T., Lebeis, S. L., Pantalone, V., Grewal, P. S., and Staton, M. E. (2019). Soil indigenous microbiome and plant genotypes cooperatively modify soybean rhizosphere microbiome assembly. *BMC Microbiol.* 19:201.

Long, X.-E., Yao, H., Huang, Y., Wei, W., and Zhu, Y.-G. (2018). Phosphate levels influence the utilisation of rice rhizodeposition carbon and the phosphate-solubilising microbial community in a paddy soil. *Soil Biol. Biochem.*118:103–114.

Lu, T., Ke, M., Peijnenburg, W. J. G. M., Zhu, Y., Zhang, M., Sun, L., et al. (2018). Investigation of rhizospheric microbial communities in wheat, barley, and two rice varieties at the seedling stage. *J. Agric. Food Chem.* 66:2645–2653.

Lundberg, D. S., Lebeis, S. L., Paredes, S. H., Yourstone, S., Gehring, J., Malfatti, S., et al. (2012). Defining the core *Arabidopsis thaliana* root microbiome. *Nature* 488:86–90.

Mahmoudi, T. R., Yu, J. M., Liu, S., Pierson, L. S., and Pierson, E. A. (2019). Drought-stress tolerance in wheat seedlings conferred by phenazine-producing rhizobacteria. *Front. Microbiol*. 10:1590.

Mahoney, A. K., Yin, C., and Hulbert, S. H. (2017). Community structure, species variation, and potential functions of rhizosphere-associated bacteria of different winter wheat (*Triticum aestivum*) cultivars. *Front. Plant Sci.* 8:132.

Maketon, C., Fortuna, A.-M., and Okubara, P. A. (2012). Cultivar-dependent transcript accumulation in wheat roots colonized by *Pseudomonas fluorescens* Q8r1-96 wild type and mutant strains. *Biol. Control* 60:216–224.

Mazzola, M., and Gu, Y.-H. (2002). Wheat genotype-specific induction of soil microbial communities suppressive to disease incited by *Rhizoctonia solani* anastomosis group (AG)-5 and AG-8. *Phytopathology* 92:1300–1307.

Mazzola, M., Funnell, D. L., and Raaijmakers, J. M. (2004). Wheat cultivar-specific selection of 2,4-diacetylphloroglucinol-producing fluorescent *Pseudomonas* species from resident soil populations. *Microb. Ecol.* 48:338–348.

Meyer, J. B., Lutz, M. P., Frapolli, M., Péchy-Tarr, M., Rochat, L., Keel, C., et al. (2010). Interplay between wheat cultivars, biocontrol pseudomonads, and soil. *Appl. Environ. Microbiol.* 76:6196–6204.

Narula, N., Kumar, V., Behl, R. K., Deubel, A., Gransee, A., and Merbach, W. (2000). Effect of P-solubilizing *Azotobacter chroococcum* on N, P, K uptake in P-responsive wheat genotypes grown under greenhouse conditions. *J. Plant Nutr. Soil Sci.* 163:393–398.

Naylor, D., DeGraaf, S., Purdom, E., and Coleman-Derr, D. (2017). Drought and host selection influence bacterial community dynamics in the grass root microbiome. *ISME J.* 11:2691–2704.

Ndour, P., Gueye, M., Barakat, M., Ortet, P., Bertrand-Huleux, M., Pablo, A., et al. (2017). Pearl millet genetic traits shape rhizobacterial diversity and modulate rhizosphere aggregation. *Front. Plant Sci.* 8:1288.

Nelson, D., Cann, I., and Mackie, R. (2010). Response of archaeal communities in the rhizosphere of maize and soybean to elevated atmospheric CO_2 concentrations. *PLoS One* 5:e15897.

Novinscak, A., and Filion, M. (2011). Effect of soil clay content on RNA isolation and on detection and quantification of bacterial gene transcripts in soil by quantitative reverse transcription-PCR. *Appl. Environ. Microbiol.* 77:6249–6252.

Offre, P., Prosser, J. I., and Nicol, G. W. (2009). Growth of ammonia-oxidizing *Archaea* in soil microcosms is inhibited by acetylene. *FEMS Microbiol. Ecol.* 70:99–108.

Okubara, P. A., and Bonsall, R. F. (2008). Accumulation of *Pseudomonas*-derived 2,4-diacetylphloroglucinol on wheat seedling roots is influenced by host cultivar. *Biol. Control* 46:322–331.

Pagnani, G., Galieni, A., Stagnari, F., Pellegrini, M., Del Gallo, M., and Pisante, M. (2020). Open field inoculation with PGPR as a strategy to manage fertilization of ancient *Triticum* genotypes. *Biol. Fertil. Soils* 56:111–124.

Paulin, M. M., Novinscak, A., St-Arnaud, M., Goyer, C., DeCoste, N. J., Privé, J.-P., et al. (2009). Transcriptional activity of antifungal metabolite-encoding genes *phlD* and *hcnBC* in *Pseudomonas* spp. using qRT-PCR. *FEMS Microbiol. Ecol.* 68:212–222.

Peiffer, J. A., Spor, A., Koren, O., Jin, Z., Tringe, S. G., Dangl, J. L., et al. (2013). Diversity and heritability of the maize rhizosphere microbiome under field conditions. *Proc. Natl. Acad. Sci. USA* 110:6548–6553.

Pérez-de-Luque, A., Tille, S., Johnson, I., Pascual-Pardo, D., Ton, J., and Cameron, D. D. (2017). The interactive effects of arbuscular mycorrhiza and plant growth-promoting rhizobacteria synergistically enhance host plant defences against pathogens. *Sci. Rep.* 7:16409.

Pérez-Jaramillo, J. E., Carrión, V. J., Bosse, M., Ferrão, L. F. V., de Hollander, M., Garcia, A. A. F., et al. (2017). Linking rhizosphere microbiome composition of wild and domesticated *Phaseolus vulgaris* to genotypic and root phenotypic traits. *ISME J.* 11:2244–2257.

Poly, F., Ranjard, L., Nazaret, S., Gourbiere, F., and Jocteur Monrozier, L. (2001). Comparison of *nifH* gene pools in soils and soil microenvironments with contrasting properties. *Appl. Environ. Microbiol.* 67:2255–2262.

Prudence, S. M. M., Newitt, J. T., Worsley, S. F., Macey, M. C., Murrell, J. C., Lehtovirta-Morley, L. E., et al. (2021). Soil, senescence and exudate utilisation: characterisation of the Paragon var. spring bread wheat root microbiome. *Environ. Microbiome* 16:12.

Ragot, S. A., Kertesz, M. A., and Bünemann, E. K. (2015). *phoD* alkaline phosphatase gene diversity in soil. *Appl. Environ. Microbiol.* 81:7281–7289.

Rascovan, N., Carbonetto, B., Perrig, D., Díaz, M., Canciani, W., Abalo, M., et al. (2016). Integrated analysis of root microbiomes of soybean and wheat from agricultural fields. *Sci. Rep.* 6:1–12.

Renoud, S., Bouffaud, M.-L., Dubost, A., Prigent-Combaret, C., Legendre, L., Moënne-Loccoz, Y., et al. (2020). Co-occurrence of rhizobacteria with nitrogen fixation and/or 1-aminocyclopropane-1-carboxylate deamination abilities in the maize rhizosphere. *FEMS Microbiol. Ecol.* 96:fiaa062.

Rilling, J. I., Acuña, J. J., Sadowsky, M. J., and Jorquera, M. A. (2018). Putative nitrogen-fixing bacteria associated with the rhizosphere and root endosphere of wheat plants grown in an andisol from southern Chile. *Front. Microbiol.* 9:2710.

Rotthauwe, J. H., Witzel, K. P., and Liesack, W. (1997). The ammonia monooxygenase structural gene *amoA* as a functional marker: molecular fine-scale analysis of natural ammonia-oxidizing populations. *Appl. Environ. Microbiol.* 63:4704–4712.

Sakurai, M., Wasaki, J., Tomizawa, Y., Shinano, T., and Osaki, M. (2008). Analysis of bacterial communities on alkaline phosphatase genes in soil supplied with organic matter. *Soil Sci. Plant Nutr.* 54:62–71.

Santhanam, R., Groten, K., Meldau, D. G., and Baldwin, I. T. (2014). Analysis of plant-bacteria interactions in their native habitat: bacterial communities associated with wild tobacco are independent of endogenous jasmonic acid levels and developmental stages. *PLoS One* 9:e94710.

Santos-Medellín, C., Edwards, J., Liechty, Z., Nguyen, B., and Sundaresan, V. (2017). Drought stress results in a compartment-specific restructuring of the rice root-associated microbiomes. *mBio* 8:e00764-17.

Siciliano, S. D., Theoret, C. M., de Freitas, J. R., Hucl, P. J., and Germida, J. J. (1998). Differences in the microbial communities associated with the roots of different cultivars of canola and wheat. *Can. J. Microbiol.* 44:844–851.

Soni, R., Suyal, D. C., Sai, S., and Goel, R. (2016). Exploration of *nifH* gene through soil metagenomes of the western Indian Himalayas. *3 Biotech.* 6:25.

Spohn, M., Zavišić, A., Nassal, P., Bergkemper, F., Schulz, S., Marhan, S., et al. (2018). Temporal variations of phosphorus uptake by soil microbial biomass and young beech trees in two forest soils with contrasting phosphorus stocks. *Soil Biol. Biochem.* 117:191–202.

Spor, A., Roucou, A., Mounier, A., Bru, D., Breuil, M.-C., Fort, F., et al. (2020). Domestication-driven changes in plant traits associated with changes in the assembly of the rhizosphere microbiota in tetraploid wheat. *Sci. Rep.* 10:12234.

Stopnisek, N., and Shade, A. (2021). Persistent microbiome members in the common bean rhizosphere: an integrated analysis of space, time, and plant genotype. *ISME J.* 15:2708–2722.

Stromberger, M. E., Abduelafez, I., Byrne, P., Canela, M. M., Elamari, A. A., Manter, D. K., et al. (2017). Genotype-specific enrichment of 1-aminocyclopropane-1-carboxylic acid deaminase-positive bacteria in winter wheat rhizospheres. *Soil Sci. Soc. Am. J.* 81:796–805.

Sugiyama, A., Ueda, Y., Zushi, T., Takase, H., and Yazaki, K. (2014). Changes in the bacterial community of soybean rhizospheres during growth in the field. *PLoS One* 9:e100709.

Svercel, M., Hamelin, J., Duffy, B., Moënne-Loccoz, Y., and Défago, G. (2010). Distribution of *Pseudomonas* populations harboring *phlD* or *hcnAB* biocontrol genes is related to depth in vineyard soils. *Soil Biol. Biochem.* 42:466–472.

Throbäck, I. N., Enwall, K., Jarvis, Å., and Hallin, S. (2004). Reassessing PCR primers targeting *nirS*, *nirK* and *nosZ* genes for community surveys of denitrifying bacteria with DGGE. *FEMS Microbiol. Ecol*. 49:401–417.

Tkacz, A., Pini, F., Turner, T. R., Bestion, E., Simmonds, J., Howell, P., et al. (2020). Agricultural selection of wheat has been shaped by plant-microbe interactions. *Front. Microbiol.* 11:132.

Treusch, A. H., Leininger, S., Kletzin, A., Schuster, S. C., Klenk, H.-P., and Schleper, C. (2005). Novel genes for nitrite reductase and Amo-related proteins indicate a role of uncultivated mesophilic *Crenarchaeota* in nitrogen cycling. *Environ. Microbiol.* 7:1985–1995.

Turner, T. R., Ramakrishnan, K., Walshaw, J., Heavens, D., Alston, M., Swarbreck, D., et al. (2013). Comparative metatranscriptomics reveals kingdom level changes in the rhizosphere microbiome of plants. *ISME J.* 7:2248–2258.

Valente, J., Gerin, F., Gouis, J. L., Moënne-Loccoz, Y., and Prigent–Combaret, C. (2020). Ancient wheat varieties have a higher ability to interact with plant growth-promoting rhizobacteria. *Plant Cell Environ.* 43:246–260.

Venieraki, A., Dimou, M., Pergalis, P., Kefalogianni, I., Chatzipavlidis, I., and Katinakis, P. (2011). The genetic diversity of culturable nitrogen-fixing bacteria in the rhizosphere of wheat. *Microb. Ecol.* 61:277–285.

Wartiainen, I., Eriksson, T., Zheng, W., and Rasmussen, U. (2008). Variation in the active diazotrophic community in rice paddy—*nifH* PCR-DGGE analysis of rhizosphere and bulk soil. *Appl. Soil Ecol.* 39:65–75.

Wu, L., Ma, K., and Lu, Y. (2009). Prevalence of betaproteobacterial sequences in *nifH* gene pools associated with roots of modern rice cultivars. *Microb. Ecol.* 57:58–68.

Yang, L., Danzberger, J., Schöler, A., and Schröder, P. (2017). Dominant groups of potentially active bacteria shared by barley seeds become less abundant in root associated microbiome. *Front. Plant Sci.* 8:1005.

Yang, M., Mavrodi, D. V., Thomashow, L. S., and Weller, D. M. (2018). Differential response of wheat cultivars to *Pseudomonas brassicacearum* and take-all decline soil. *Phytopathology* 108:1363–1372.

Zhang, C., Lin, Y., Tian, X., Xu, Q., Chen, Z., and Lin, W. (2017). Tobacco bacterial wilt suppression with biochar soil addition associates to improved soil physiochemical properties and increased rhizosphere bacteria abundance. *Appl. Soil Ecol.* 112:90–96.

Zuo, S., Li, X., Ma, Y., and Yang, S. (2014). Soil microbes are linked to the allelopathic potential of different wheat genotypes. *Plant Soil* 378:49–58.

Table S1. Analysis of bacterial, archaeal and fungal communities in rhizosphere soil (RS) or root endosphere (RE) of wheats and non-wheat plants. Primer pairs used are 515F/806R (a), 341F/806R (b), 27F/518R (c), 515F/907R (d), 519F/926R (e), 341F/785R (f), SDBact-0008-a-S-16F/SDBact-0343-a-A-15R (g), (799F/1193R (h), 114F/1392R (i), 8F/533R (j), 27F/1492R (k),1070F/1492R (l), or 5'-CCTACGGGNGGCWGCAG-3'/5'-GACTACHVGGGTATCTAATCC-3' (m) for bacteria (B), A0349F/A0519R (n), 524F/Arch958modR (o), Arch-amoF2/arch-amoAR (p), or 519F/958R (q) for archaea (A), and ITS1/ITS2 (r), ITS1/ITS4 (s), ITS3/ITS4 (t), ITS9/ITS4 (u), ITS5/ITS2 (v), or LROR/LR3 (w) for fungi (F). Each condition listed is a particular combination of one soil × one variety × one growth stage, for which the root compartment(s) studied and metabarcoding specifics are indicated. List of references is available in Supplementary data S1.

Plant host	Geographic location[2] and soil	Plant variety (and phenology)	Compartment[1]	Microbiota	Sequencing	Reference
**Wheats**						
T. aestivum						
Condition 1	Bawburgh (UK), grassland soil (Luvisol)	Paragon (heading/flowering stage)	RS and RE	B (a), F (q)	Illumina MiSeq	Tkacz et al. 2020
Condition 2	Bawburgh (UK), crop soil (Luvisol)	Paragon (heading stage)	RS and RE	B (b), A (n)	Illumina MiSeq	Prudence et al. 2021
Condition 3	Pullman (WS), crop soil (Argixerolls)	Madsen (ripening stage)	RS	B (c)	Illumina MiSeq	Mahoney et al. 2017
Condition 4	Pullman (WS), crop soil (Argixerolls)	PI561725 (ripening stage)	RS	B (c)	Illumina MiSeq	Mahoney et al. 2017
Condition 5	Kirksville (MO), clay (Hapludalfs) mixed with sterile potting soil	GSTR 11562 (heading stage)	RE	F (r)	Pyrosequencing	Bokati et al. 2016
Condition 6	Norwich (UK), crop soil (not described)	Paragon (not specified)	RS	B (c)	Pyrosequencing	Turner et al. 2013
Condition 7	Northern China, crop soils (Cambisols, Luvisols, Calcisols)	Not specified (filling stage)	RS	B (a)	Illumina MiSeq	Fan et al. 2018
Condition 8	Hangzhou (China), paddy soil (not described)	Not specified (seedling stage)	RS	F (not specified)	Illumina HiSeq	Lu et al. 2018
Condition 9	Napier (South Africa), crop soil (not described)	SST88 (not specified)	RS	F (r)	Illumina MiSeq	Gqozo et al. 2020
Condition 10	Pretoria (South Africa), crop soil (not described)	Kariega (not specified)	RS	F (r)	Illumina MiSeq	Gqozo et al. 2020
Condition 11	Bethlehem (South Africa), crop soil (not described)	Eland (not specified)	RS	F (r)	Illumina MiSeq	Gqozo et al. 2020
T. durum						
Condition 1	Bawburgh (UK), grassland soil (Luvisol)	ENT392 (heading/flowering stage)	RS and RE	B (d), A (n), F (t)	Illumina MiSeq	Tkacz et al. 2020
T. dicoccoides						
Condition 1	Bawburgh (UK), grassland soil (Luvisol)	Biensur (heading/flowering stage)	RS and RE	B (d), A (n), F (t)	Illumina MiSeq	Tkacz et al. 2020
A.tauschii						
Condition 1	Bawburgh (UK), grassland soil (Luvisol)	DIC70 (heading/flowering stage)	RS and RE	B (d), A (n), F (t)	Illumina MiSeq	Tkacz et al. 2020
**Barley**						
Hordeum vulgare						
Condition 1	Golm (Germany), sandy experimental soil (not described)	HID369 (not specified)	RS	B (a)	Pyrosequencing	Bulgarelli et al. 2015
Condition 2	Scheyern (Germany), sandy crop soil (Cambisol)	Alexis (booting stage)	RE	B (g)	Illumina MiSeq	Yang et al. 2017

Condition 3	Albany (CA), silty loam crop soil (Cambisol)	Ethiopian (pre-flowering stage)	RE	B (f)	Illumina MiSeq	Naylor et al. 2017
**Oat**						
Avena strigosa						
Condition 1	Norwich (UK), crop soil (not described)	S75 (heading stage)	RS	B (not specified)	Metatranscriptomics	Turner et al. 2013
Avena sativa						
Condition 2	Albany (NY), silty loam crop soil (Cambisol)	Monico (pre-flowering stage)	RE	B (f)	Illumina MiSeq	Naylor et al. 2017
**Millet**						
Pennisetum glaucum						
Condition 1	Bambey (Senegal), sandy crop soil (Arenosol)	L118 (vegetative stage)	RS	B (a)	Ion torrent	Ndour et al. 2017
Condition 2	Bambey (Senegal), sandy crop soil (Arenosol)	L14 (vegetative stage)	RS	B (a)	Ion torrent	Ndour et al. 2017
Seratia italica						
Condition 1	Changzhi (China), sandy clay loam natural soil (not described)	Changnong35 (flowering stage)	RS	B (a)	Illumina MiSeq	Han et al. 2017
Condition 2	Changzhi (China), sandy clay loam natural soil (not described)	Jingu21 (flowering stage)	RS	B (a)	Illumina MiSeq	Han et al. 2017
**Sorghum**						
Sorghum bicolor						
Condition 1	Albany (NY), silty loam crop soil (Cambisol)	Atx642 (pre-flowering stage)	RE	B (f)	Illumina MiSeq	Naylor et al. 2017
Condition 2	Albany (NY), silty loam crop soil (Cambisol)	BTx623 (pre-flowering stage)	RE	B (f)	Illumina MiSeq	Naylor et al. 2017
**Maize**						
Zea mays						
Condition 1	Champaign (IL), fine-silty crop soil (Endoaquoll)	34B43 (post-emergence stage)	RS	B (e)	Pyrosequencing	Li et al. 2014
Condition 2	Lansing (NY), silt loam crop soil (Endoaquepts)	B73 (flowering stage)	RS	B (a)	Pyrosequencing	Peiffer et al. 2013
Condition 3	Champaign (IL), fine-silty crop soil (Endoaquoll)	34B43 (heading stage)	RS	A (o)	Pyrosequencing	Nelson et al. 2010
Condition 4	Davis (CA), nutrient-depleted crop soil (Xerorthents or Haploxeralfs)	3382 (stem extension stage)	RS	B (a)	Illumina MiSeq	Brisson et al. 2019
Condition 5	Albany (NY), silty loam crop soil (Cambisol)	M017 (pre-flowering stage)	RE	B (f), A (c), F (u)	Illumina MiSeq	Naylor et al. 2017
**Rice**						
Oryza sativa						
Condition 1	Davis (CA), crop soil (not described)	M206 (vegetative stage)	RS and RE	B (a), F (r)	Illumina MiSeq	Santos-Medellin et al. 2017

Condition 2	Davis (CA), crop soil (not described)	M104 (vegetative stage)	RS and RE	B (a)	Illumina MiSeq	Edwards et al. 2015
**Tobacco**						
Nicotinia tabacum						
Condition 1	Nanping (China), crop soil (not described)	K326 (ripening stage)	RS	B (j)	Pyrosequencing	Zhang et al. 2017
Nicotiana attenuata						
Condition 2	Great Basin Desert (UT), crop soil (Petrocalcids)	Watson (flowering stage)	RE	B (a)	Pyrosequencing	Santhanam et al. 2014
**Tomato**						
Solanum lycopersicum						
Condition 1	Yecheon (South Korea), greenhouse soil (not described)	Alexander (ripening stage)	RS and RE	B (h), A (p), F (t)	Illumina MiSeq	Lee et al. 2019
Condition 2	Busan (Korea), silt loam crop soil (not described)	Moneymaker (flowering stage)	RS	B (c)	Illumina MiSeq	Kwak et al. 2018
Condition 3	Busan (Korea), silt loam crop soil (not described)	Hawaii 7996 (flowering stage)	RS	B (c)	Illumina MiSeq	Kwak et al. 2018
Condition 4	Barka (Oman), sandy crop soil (not described)	Not specified (not specified)	RS	F (s)	Pyrosequencing	Kazerooni et al. 2017
**Bean**						
Phaseolus vulgaris						
Condition 1	Othello (WS), crop soil (not described)	Eclipse (flowering stage)	RS	B (a), A (a), F(r)	Illumina MiSeq	Stopnisek and Shade 2021
Condition 2	El Carmen de Viboral (Colombia), crop soil (not described)	G14947 (V4 pre-flowering stage)	RS	B (m)	Illumina MiSeq	Perez-Jaramillo et al. 2017
Condition 3	El Carmen de Viboral (Colombia), crop soil (not described)	Not specified (flowering stage)	RS	B (m)	Illumina MiSeq	Perez-Jaramillo et al. 2017
**Soybean**						
Glycine max						
Condition 1	Knoxville (TN), crop soil (Paleudults)	Williams (flowering stage)	RS	B (f)	Illumina MiSeq	Liu et al. 2019
Condition 2	Villages in Buenos Aires Region (Argentina), pampa soils (Hapludolls)	Williams 82 (not specified)	RS and RE	B (a)	Pyrosequencing	Rascovan et al. 2016
Condition 3	Kyoto (Japan), crop soil (not described)	Fukujishi (flowering stage)	RS	B (c)	Pyrosequencing	Sugiyama et al. 2014
Condition 4	Changchun (China), crop soil (not described)	Heilong48 (ripening stage)	RS	F (s)	Pyrosequencing	Bai et al. 2015
Condition 5	Jiaxiang (China), crop soil (not described)	Shannong 20 (flowering stage)	RS	F (v)	Illumina MiSeq	Han et al. 2017
**Arabidopsis**						
Arabidopsis thaliana						
Condition 1	Benton Harbor (MI), natural soil (not described)	Pna-10 (bolting stage)	RS	B (c)	Pyrosequencing	Chaparro et al. 2014
Condition 2	Chapel Hill (NC), crop soil mixed with sterile soil (not described)	Col-0 (flowering stage)	RS and RE	B (i)	Pyrosequencing	Lundberg et al. 2012

Condition 3	Cologne (Germany), crop loam soil (not described)	Landsberg erecta (flowering stage)	RS and RE	B (k)	Pyrosequencing	Bulgarelli et al. 2012
Condition 4	Cologne (Germany), crop loam soil (not described)	Shakdara (flowering stage)	RS and RE	B (h)	Pyrosequencing	Bulgarelli et al. 2012
**Poplar**						
Populus deltoides						
Condition 1	Buffalo Valley (TN), sandy loam natural soil (not described)	Native ecotype (mature tree)	RS and RE	B (a), F (w)	Pyrosequencing	Gottel et al. 2013
Condition 2	Smith County (TN), natural soils (not described)	Not specified (mature tree)	RS and RE	B (l), F (w)	Pyrosequencing	Shakya et al. 2013

[1] For all studies on the root endosphere compartment, roots were surface-sterilized before macerating root tissues.

[2] Abbreviations were used to designate United Kingdom (UK), Washington State (WS), Missouri (MO), New-York State (NY), Illinois (IL), Michigan (MI), California (CA), Utah (UT), Tennessee (TN), and North Carolina (NC).

Table S2. Molecular tools available for direct analysis of root-associated microbial functional groups. Asterisks indicate studies that used the primers in the case of wheat. List of references is available in Supplementary data S1.

Microbial function	Gene marker	Method	Primers (and amplicon size)	Primer reference	Examples of studies where method was used
**Biotic interactions**					
DAPG synthesis	*phlD*		Phl2a/Phl2q (127 bp)	Paulin *et al.* 2009	
		qPCR	B2BF/B2BR3 (319 bp)	Almario *et al.* 2013	Hu *et al.* 2016
			PhlD_65F_DEG/PhlD_236R_DEG	Imperiali *et al.* 2017	Dennert *et al.* 2018
		qRT-PCR	Phl2a/Phl2q (127 bp)	Paulin *et al.* 2009	
			phlD-fwd/phlD-rev (58 bp)	DeCoste *et al.* 2011	Gadkar and Filion 2013
Hydrogen cyanide synthesis	*hcnAB*	PCR	PM2/PM726R (570 bp)	Svercel *et al.* 2007	
	hcnBC	qPCR	HCNn/Acb (233 bp)	Paulin *et al.* 2009	
		qRT-PCR	HCNn/Acb (233 bp)	Paulin *et al.* 2009	
	hcnC	qRT-PCR	hcnC-fwd/hcnC-rev (60 bp)	DeCoste *et al.* 2011	Novinscak et Filion 2011; Gadkar and Filion 2013
Phenazine synthesis	*phzF*	qPCR	PhzF_2Fm/PhzF_2Rm	Imperiali *et al.* 2017	Dennert *et al.* 2018
ACC deaminase activity	*acdS*	qPCR	acdSF5/acdSR8 (133 bp)	Bouffaud *et al.* 2018*	Renoud *et al.* 2020
		qRT-PCR	acdSF5/acdSR8 (133 bp)	Bouffaud *et al.* 2018*	Renoud *et al.* 2020

		Illumina MiSeq	acdSF5/acdSR8 (133 bp)	Bouffaud *et al.* 2018*	Renoud *et al.* 2020
**Biogeochemical cycles**					
N_2 fixation	*nifH*	PCR	polF/polR (360 bp)	Poly *et al.* 2001	Wartiainen *et al.* 2008
		qPCR	polF/polR (360 bp)		Bouffaud *et al.* 2016; Renoud *et al.* 2020
		RT-PCR	nifH-g1-forB/ nifH-g1-rev (371 bp)	Bürgmann *et al.* 2003	
		qPCR	nifH-g1-forB/ nifH-g1-rev (371 bp)		Rilling *et al.* 2018*
		qRT-PCR	polF/polR (360 bp)		Bouffaud *et al.* 2016
		Illumina MiSeq	polF/polR (360 bp)		Renoud *et al.* 2020
		DNA cloning-sequencing	polF/polR (360 bp)		Soni *et al.* 2016
Nitrification	Archaeal *amoA*	qPCR, qRT-PCR	*amo*196F/*amo*277R	Treusch *et al.* 2005	Leininger *et al.* 2006
		qPCR	CrenamoA1-165f/CrenamoA1-309r (226 bp)	Offre *et al.* 2009	
		qPCR	CrenamoA2-165f/CrenamoA2-390r (226 bp)	Offre *et al.* 2009	
		DNA cloning-sequencing	*amo*A19F/*amo*A643R	Treusch *et al.* 2005	Leininger *et al.* 2006
	Bacterial *amoA*	PCR	*amo*A-1F/*amo*A-2R (491 bp)	Rotthauwe *et al.* 1997	
		qPCR	*amo*A-1F/*amo*A-2R (491 bp)		Leininger *et al.* 2006; Offre *et al.* 2009
Denitrification	*nirK,*	PCR	nirK876/nirK1040 (165 bp)	Henry *et al.* 2004	
		qRT-PCR	nirK876/nirK1040 (165 bp)		Achouak *et al.* 2019*

	nirS	PCR	nirSCd3Af/nirSR3cd	Throbäck *et al.* 2004	
		qRT-PCR	nirSCd3Af/nirSR3cd		Achouak *et al.* 2019*
	nosZI	qPCR	nosZ1F/noZIR (259 bp)	Henry *et al.* 2006	Spor *et al.* 2020*
		qPCR	nosZ1F/noZIR (259 bp)	Henry *et al.* 2006	Spor *et al.* 2020*
	nosZII	qPCR	noZ2F/nos2R (267 bp)	Henry *et al.* 2006	Spor *et al.* 2020*
		qPCR	noZ2F/nos2R (267 bp)	Henry *et al.* 2006	Spor *et al.* 2020*
Phosphorus mineralization	*phoD*	PCR	ALPS-F730/ALPS-R1101 (371 bp)	Sakurai *et al.* 2008	Wu *et al.* 2009
		qPCR	ALPS-F730/ALPS-R1101 (371 bp)		Wan *et al.* 2020
		Illumina MiSeq	*phoD*-FW/*phoD*-RW (208 bp)	Bergkemper *et al.* 2016	
		Illumina MiSeq	*phoD*-F733/*phoD*-R1083 (350 bp)	Ragot *et al*. 2015	Long *et al.* 2018
		qPCR	*phoD*-F733/*phoD*-R1083 (350 bp)		Chen *et al.* 2017
	phoN	Illumina MiSeq	*phoN*-FW/*phoN*-RW (159 bp)	Bergkemper *et al.* 2016	
		qPCR	*phoN*-FW/*phoN*-RW (159 bp)		Spohn *et al.* 2018
	appA	Illumina MiSeq	*appA*-FW/*appA*-RW (375 bp)	Bergkemper *et al.* 2016	
	phnX	Illumina MiSeq	*phnX*-FW/*phnX*-RW (147 bp)	Bergkemper *et al.* 2016	
Phosphorus solubilization	*Gcd*	Illumina MiSeq	*gcd*-FW/*gcd*-RW (330 bp)	Bergkemper *et al.* 2016	Li *et al*. 2017
	pqqC	DNA cloning-sequencing	PqqCr1/pqqCf1 (546 bp)	Meyer *et al.* 2010	
